# Mechanical Properties of Micro/Nanocellulose-Filled Epoxy Sheets at Subzero and Elevated Temperatures

**DOI:** 10.1021/acsomega.5c09299

**Published:** 2025-12-17

**Authors:** Pallavi Gulipalli, Chandra Babu Mallineni, Ramesh Adusumalli, Ramendra Kishor Pal

**Affiliations:** Department of Chemical Engineering, 209298 Birla Institute of Technology and Science, Pilani, Hyderabad Campus, Jawahar Nagar, Kapra Mandal, Medchal District, Hyderabad, Telangana 500078, India

## Abstract

Due to their low cost and 100% biodegradability, nanocellulose fibrils derived from wood can serve as a filler in polymer sheets. In this study, micro/nanocellulose fibrils were obtained after Lab Valley beater (LVB) and Super masscolloider (SMC) refining. The LVB refining was carried out for 20 min at 0 clearance (LVB_0_20), and SMC refining was carried out for 60 min at 0.1 and 0 clearances (SMC_0.1_60 and SMC_0_60), and nonwoven sheets were fabricated using a vacuum filtration. These micro/nanocellulose-filled epoxy nonwoven sheets were processed by the hand layup/vacuum bagging and cured at 160 °C for 3 h. The tensile properties of filled epoxy sheets were measured at −20 °C, 23 °C, and 80 °C, and it was found that the SMC_0_60_epoxy sheet revealed high modulus at all temperatures compared to other sheets, but its strength and work-to-fracture values are found to be low at 80 °C compared to −20 °C. SMC_epoxy sheets have high tensile strength compared to LVB_epoxy sheets due to the presence of nanocellulose fibrils. The tensile fractography was carried out using SEM. Filled epoxy sheets revealed 45–70% higher thermal conductivity and slightly lower weight loss at 430 °C compared to epoxy, making these sheets useful in developing optimum strength and partially biodegradable thermal packaging components.

## Graphical abstract

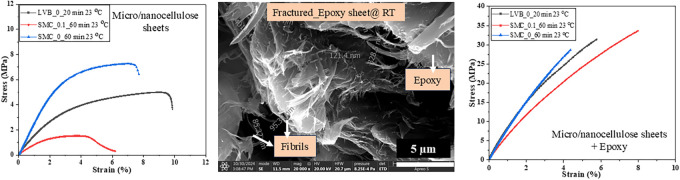

## 1 Introduction

In recent years, the quest for sustainable materials has driven researchers and industries toward innovative solutions that balance performance with environmental responsibility. Among these, cellulose fiber-based materials are gaining significant attention as a promising alternative to synthetic materials due to their availability, cost-effectiveness, ease of manufacture, optimum strength, and biodegradability.^1^ Cellulose fiber-based fillers are used instead of glass, carbon, silica, Kevlar, basalt, and metal fillers to reduce the density of the specimens. Cellulose fibers have a lower density (1.2–1.6 g/cm^3^) than glass fibers (2.4 g/cm^3^), allowing them to produce lightweight materials. As a result, there is a growing demand for cellulose fiber-based materials in commercial applications across a variety of industries.^2^ Plant biomass is one of the most abundant and naturally available cellulose materials, which has captured significant attention in recent years.^3^ Derived from lignocellulosic materials such as wood, flax, sisal, and agricultural residues, this cellulose material is not only renewable but also biodegradable, making it a less polluting and environmentally friendly choice because it is considered CO_2_ neutral.^4^

The mechanical process can cause irreversible alterations in cellulosic fibers and increase their bonding potential.^5,6^ Among the latest advancements, micro- and nanocellulose fibers stand out as revolutionary materials. Extracted from plant-based cellulose, these fibrils offer remarkable surface area, low density, and biodegradability, making them highly suitable for use as fillers.^7^ Researchers have emphasized the use of natural fibers as reinforcement or filler materials in polymer composites. Generally, flax, jute, kenaf, hemp, sugar palm, sisal, oil palm, pineapple leaf, and henequen fibers or nanocellulose (NC) fillers derived from natural fibers are mixed with polymer matrices to produce green composites. Typically, polymer matrices are classified into petroleum-based forms (vinyl ester, epoxy, polyethylene, etc.). These are the most extensively used polymers for manufacturing biofiber-reinforced composites.^8,9^ Microcellulose fibers, typically ranging from 5 to 50 μm, and nanocellulose, with its nanosized fibrils, exhibit unique structural properties.^10^ Their optimum length, diameter, and high aspect ratio allow for effective dispersion in the polymer matrix, enhancing structural integrity and interfacial bonding with the epoxy resin.^11^ In case of no-load bearing components, cellulose fiber-filled epoxy components can be used as sustainable materials.^12^

Epoxy resins are widely used in structural composites due to their ease of processing (requiring low pressure because of low viscosity and moderate cure temperatures) as well as their excellent thermal stability and mechanical properties. A framework to study the effects of environmental factors on composite performance by characterizing neat epoxy resin, interfaces, and glass—epoxy composites was reported.^13^ From this study, neat epoxy (0% fiber) exhibited a tensile strength of 34.31 MPa, which increased to 54.79 MPa with 30% curaua fiber reinforcement and to 71.91 MPa with 30% glass fiber reinforcement. This study demonstrated that synthetic fibers possess higher tensile strength compared to natural fibers, although they are nonbiodegradable.^14^ A study of the Jute epoxy composite tensile strength found it to be 12.46 MPa.^15^ Nanocellulose combined with epoxy showed tensile strength of 24.63 MPa.^16^ Epoxy matrix composites reinforced with woven bamboo-cotton fibers, where the number of layers ranged from 8 to 14, revealed the tensile strength of 36 to 47 MPa, respectively. The tensile strength increased with fiber content, reaching a maximum of 56.07 MPa for the 12-layer composite before decreasing.^17^ The lyocell-epoxy system showed that lyocell fibers exhibited higher interfacial shear strength (IFSS) with epoxy than with polypropylene, while ramie cellulose fibers showed superior adhesion due to their rough surface compared to lyocell fibers. Maleic anhydride modification of lyocell further enhanced IFSS with PP by 2-fold.^18^ Natural fibers limit the properties of reinforced composites due to their differences in comparison to synthetic fibers and their hydrophobicity.

Wood fibers, a key source of cellulose, bring unique advantages over synthetic polymers. Their structure, composed of cellulose, hemicellulose, and lignin, facilitates effective interactions with epoxy matrices, resulting in superior stress transfer and enhanced adhesion at the fiber–matrix interface.^19^ Phenol-formaldehyde resin combined with nanofiber cellulose improved tensile properties.^5,6^ Epoxy resin, a widely used thermoset polymer, is particularly compatible with natural fibers. Its chemical resistance, durability, and strong interfacial interactions via hydrogen bonding make it an excellent matrix material.^11^ This compatibility enhances the thermal properties of the epoxy, as the load is effectively transferred between the epoxy and the filler material. The combination of wood-derived cellulose and epoxy resin yields a new material with improved durability, thermal stability, and weather resistance. Despite these advancements, few challenges persist in the promotion of nanocellulose-filled epoxy sheets. Issues like moisture absorption and fiber–matrix compatibility require further exploration to make it usable in new packaging applications. However, the potential of these nanocellulose-filled epoxy sheets is undeniable, with applications ranging from packaging to furniture.^20^

Micro/nanocellulose fiber-based sheets (nondensified) do not improve the strength/stiffness of epoxy resins; they are added as fillers to improve the thermal stability of epoxy resins. The second reason to add these micro/nanocellulose fiber-based nonwoven sheets as fillers is their 100% biodegradability. In this study, we examined the tensile properties of micro/nanocellulose-filled epoxy material under varying temperatures (−20 °C, 23 °C (RT), and 80 °C), thermal properties using thermal conductivity and TGA, water absorption, and contact angle measurements to check the wettability of micro/nanocellulose-filled epoxy sheets. These sheets retain most of their mechanical properties at elevated temperatures and exhibit a high thermal stability. The water absorptivity is less (Avg of 3% at 60 °C, 90–98% rh). By examining the performance of these materials under different conditions, we aim to contribute to the growing body of knowledge on sustainable material development and unlock new opportunities for their application in the packaging segment.

## 2 Materials and Methods

### 2.1 Materials

Wood is used as a raw material to make pulp, and this pulp is used as a raw material to make micro/nanocellulose fibers. Pulp slurry consisting of micro/nanocellulose fibers is used to make nonwoven sheets, which are used as filler material. For the current work, epoxy resins (Epofine 1555 as resin + Finehard (FH)-5200 as hardener) were supplied by M/s. FFOPL, Mumbai, India. It is a liquid epoxy resin that consists of bisphenol A as the main compound, having a viscosity of 14,000–18,000 cP, and a specific gravity of 1.2 g/cm^3^ @ 25 °C. Liquid nitrogen is used for testing micro/nanocellulose sheets (nonwoven mats) at −5 °C and micro/nanocellulose-filled epoxy sheets at −20 °C.

### 2.2 Processing of Micro/Nanocellulose Fibers Using LVB and SMC

Wood chips were kraft cooked at 165 °C for 3 h to produce delignified pulp and subsequently subjected to elemental chlorine-free (ECF) bleaching. As a result, the bleached pulp has a negligible lignin content and is rich in cellulose. This bleached cellulose pulp was converted into micro- and nanocellulose fibers through extensive refining, which was carried out using a Lab Valley beater (LVB) and a Super masscolloider (SMC). LVB refining was carried out for 20 min (LVB_20 min), and only microcellulose fibers were obtained, as shown in Table 1 and Figure 1.

**Figure 1:**
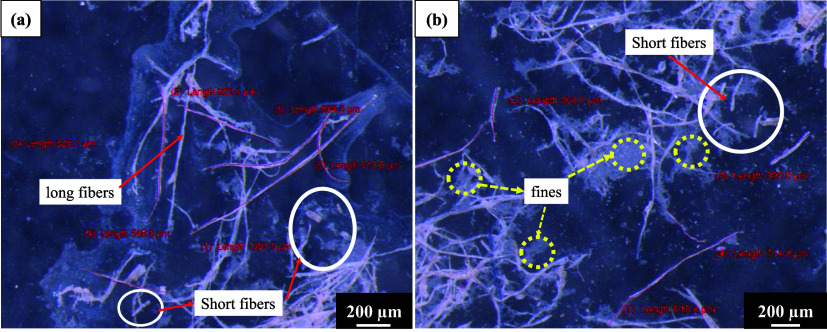
Stereomicroscopy images of bleached pulp slurry consisting of LVB_20 min microcellulose fibers (a, b).

**Table 1:** Bleached Pulp Properties After Refining of 20 min (LVB) and 60 min (SMC)

refining of bleached pulp slurry (LVB/SMC)	length (μm)	diameter (μm)	aspect ratio (l/d) of fibers	brightness (%)
LVB_20 min	506 ± 205	24.37 ± 3.5	20.1	71.1
SMC_0.1_60 min	1300 ± 159	5.53 ± 1.1	236	82.0
SMC_0_60 min	765 ± 200	0.33 ± 0.2	2353	80.5

The SMC refining was carried out for 10 min at each clearance level, starting at a clearance of 2 mm with a rotational speed of 1000 rpm and gradually reducing the clearance to 2.0, 1.0, finally to 0.4, 0.1, 0.01, and 0, where the pulp was refined for 60 min (SMC_0.1_60, SMC_0.01_60, and SMC_0_60 min). For SMC_0 clearance, 150 g of bleached sapwood pulp was soaked in 6 L of water overnight. The procedure starts with adding 6.6 L of water to the slurry at 1.5 mm clearance, followed by incremental additions of 2 L at 1 mm clearance, 4 L at 0.5 mm clearance, 5 L at 0.1 mm clearance, and 2 L at 0.01 mm clearance. Water is being added to avoid clogging while grinding.^21^ After reducing the clearance level to zero, the refining process was continued for 60 min (SMC_0_60 min), wherein micro/nanocellulose fibers were successfully produced through the SMC refining process, confirming the efficiency of this method in breaking down microfibers to micro and nanocellulose fibrils, as shown in Figure 2a,b.^10^ The unique arrangement of grooves within the grinders in SMC refining allows the formation of microcellulose fibers (Figure 2a,b) and nanocellulose fibrils, as shown in Figure 2c,d and Table 1.^10,22^

**Figure 2:**
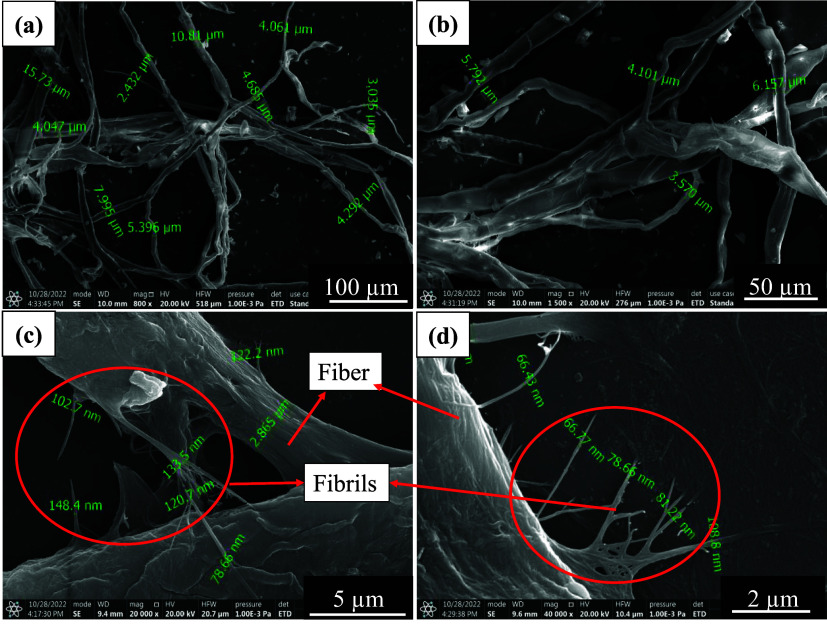
SEM images of bleached pulp slurry consisting of SMC_0.4_60 min refined fibers diameters in micrometers, (a, b); SMC_0_60 min refined fibers (diameters in microns; c), and note the fibril diameters in nanometers (c, d).

### 2.3 Processing of Nonwoven Sheets Using Micro/Nanocellulose Fibers

A handsheet former, equipped with Whatman filter paper with a 10 μm pore size, was employed to make micro/nanocellulose sheets. During the process, 300 mL of thoroughly mixed and refined pulp fiber slurry was poured through the equipment’s feed inlet, and sheets of LVB_0_20 min (Figure 3a,b), SMC_0.1_60 min (Figure 3c,d), and SMC_0_60 min (Figure 3e,f) were successfully processed. To remove excess unbound water from the wet sheet, vacuum was applied, and the pressure release valve was opened, thereby creating a uniform sheet. Later, these sheets were densified with a heavy roller and placed in a hot air oven running at 60 °C for 2 h to remove the bound moisture effectively. Upon completion of the drying process, the sheets were stored in plastic bags.^10,23^

**Figure 3:**
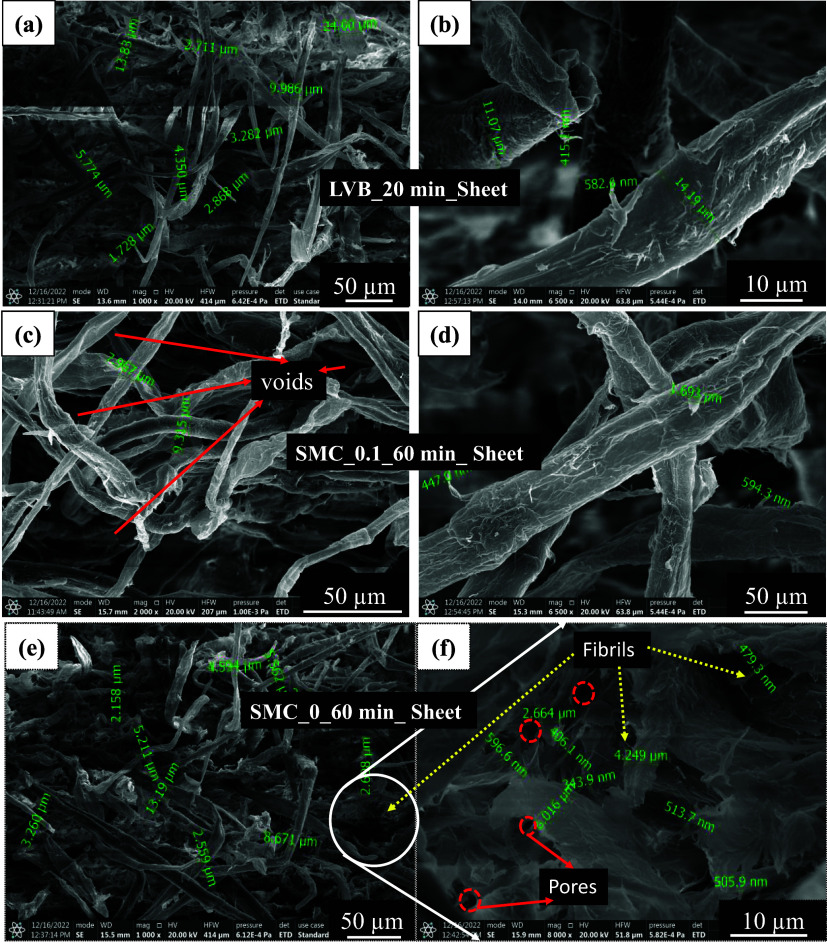
SEM images of the LVB_20 min sheet, which consists of microcellulose fibers (a). Note the fibril diameters in nm (b); SMC_0.1_60 min sheet, which consists of microcellulose fibers (c)—note the fibril diameters in nm (d); SMC_0_60 min refined fibers (diameters in microns as shown in e), and note the fibril diameters in nanometers (f).

### 2.4 Manufacturing of Micro/Nanocellulose-Filled Epoxy Sheets

Partially biodegradable sheets were prepared by using micro/nanocellulose sheets as filler and epoxy as matrix, with the hand-laying method followed by vacuum oven curing. An epoxy matrix with a resin (Epofine 1555) to hardener (Finehard (FH) 5200) mixing ratio of 100:27 was prepared. Prior to micro/nanocellulose-filled epoxy sheet preparation, an iron heating plate/mold was used for the hand layup process. It was cleaned with acetone, and a layer of waxpol was applied to ease the removal of components once the curing cycle is completed. Resin was heated at 45 °C to reduce its viscosity, and then, the hardener was added to the preheated resin (300–400 cP) to ensure easy and uniform mixing. Epoxy was poured and spread evenly on both sides (top and bottom) of the micro/nanocellulose sheet with the help of a roller for better impregnation. 1–2 epoxy impregnated nonwoven sheets were placed in a stack, followed by a peel ply to achieve an overall surface finish for components, as shown in Figure 4. Following that, a breather cloth was used to trap and hold excess epoxy during the vacuum process, and vacuum bagging was used to eliminate air bubbles, create high-pressure compaction, and ensure a tight bond between all layers. Vacuum bag film is sealed with sealant tape, covering the mold area. The mold was kept in a vacuum oven at 120 °C—3 h, followed by 160 °C—3 h with 2–3 bar pressure throughout the T-t cycle. Components were demolded and found to have no macrovoids. Three different types of micro/nanocellulose fiber-filled epoxy sheets were prepared (LVB_0_20 min_epoxy, 1–1.2 mm thick, resin content of 55 wt %; SMC_0.1_60 min_epoxy, 1.8–2 mm thick, resin content of 65 wt %; and SMC_0_60 min_epoxy, 1–1.2 mm thick, resin content of 55 wt %). The nonwoven sheets were not pressed during manufacturing; hence, the term ‘filler’ is used instead of ‘reinforcement’ to describe these sheets. In one study, pressure of 100 and 10 MPa was used to densify the sheets, wherein the resin contents were found to be low (below 30 wt %).^6,24^ As per ASTM 3039, samples were cut using a high-speed hand cutter, and mechanical tests were performed at subzero (−20 °C) and elevated temperatures (80 °C).

**Figure 4:**
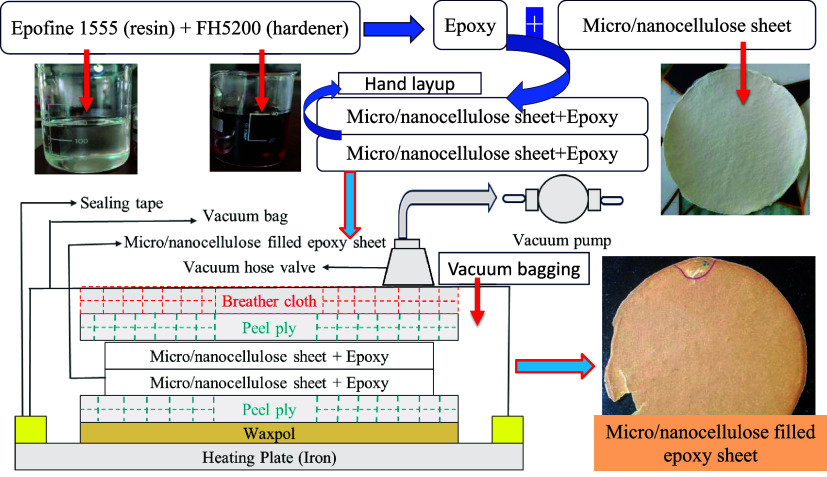
Manufacturing of micro/nanocellulose-filled epoxy sheets using the hand layup, followed by the vacuum bagging and curing at 120 °C—3 h, followed by 160 °C—3 h.

### 2.5 Tensile Testing of Nonwoven Sheets, Epoxy Specimens, and Micro/Nanocellulose Fiber-Filled Epoxy Sheets

The tensile testing of both micro/nanocellulose sheets^22^ and micro/nanocellulose-filled epoxy samples was conducted using a Zwick/Roell universal testing machine (UTM) equipped with a 5 kN load cell as per ASTM D3039. This UTM system includes an environmental temperature chamber (as shown in Figure 5), which enables controlled testing across a temperature range from −70 to 200 °C. Tensile tests of micro/nanocellulose-filled epoxy samples (LVB_0_20 min_epoxy, SMC_0.1_60 min_epoxy, and SMC_0_60 min_epoxy) were conducted at three distinct temperatures of −20 °C, room temperature (RT-23 °C), and 80 °C. The tensile testing of micro/nanocellulose sheets was also carried out at three different temperatures of −5 °C, RT-23 °C, and 65 °C. To understand the influence of relative humidity (RH), micro/nanocellulose-filled epoxy sheets were also tested at 98% RH.

**Figure 5:**
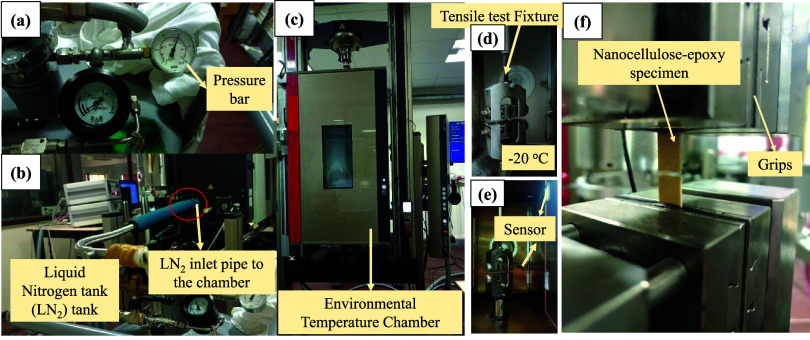
Environmental chamber setup used for tensile testing of micro/nanocellulose-filled epoxy sheets at −20 °C, 23 °C, and 80 °C: (a) pressure bar and gauge, (b) liquid nitrogen (LN_2_) tank with inlet line to the chamber, (c) environmental temperature chamber mounted on the universal testing machine, (d) tensile test fixture inside the chamber, (e) temperature sensor positioned near the specimen, and (f) nanocellulose-epoxy tensile specimen clamped between the grips.

The environmental temperature chamber inside the UTM consists of two sensors: one measures the chamber’s internal temperature, while the other keeps track of the specimen’s temperature (Figure 5). For tests conducted at elevated temperatures, hot air circulates consistently within the chamber, creating stable conditions at 65 or 80 °C. For experiments at subzero temperatures, liquid nitrogen (cooling agent) is being pumped to the environmental chamber at a controlled pressure of 1.4 bar, as shown in Figure 5, and is kept circulating to maintain the low-temperature environment needed to perform tensile tests at −5 or −20 °C. Prior to each test, all specimens were preconditioned in the environmental chamber for a minimum of 5 min. As per the ASTM D3039, the test was conducted with a constant crosshead speed of 1 mm/min. The gauge length for micro/nanocellulose sheet testing is 25 mm, and for a micro/nanocellulose-filled epoxy sheet, it is reduced to 13 mm. Specimen dimensions of 60 mm in length, 10–14 mm in width, and a thickness ranging from 0.6 mm to 2 mm were used for the tensile test.

Micro/nanocellulose sheets are lightweight but delicate because the sheets are not fully densified, making them prone to slippage or damage when held with metal grips under tensile loading. Hence, rubber grips were used in this study. These grips are soft and have a nonslip surface, which securely holds the sheets and prevents tear or damage. Additionally, rubber’s natural elasticity helps in spreading the gripping force evenly, reducing stress at the edges and keeping the micro/nanocellulose sheet intact during testing. Since the epoxy liquid flows easily through the sheet, the surface of the epoxy sheet is smooth, and it can increase the chance of slippage in the case of rubber grips. To prevent this, metal grips were used instead of rubber grips for tensile testing of filled epoxy sheets to ensure secure clamping, precision, and accuracy in measurement. Strain gauges were not used in the tensile testing of nanocellulose sheets and filled epoxy sheets due to the thin and smooth surfaces of these materials, making it difficult to attach the strain gauges. Instead, strain was calculated using a crosshead displacement measurement, which provides reliable strain data without altering the specimen.

### 2.6 Microscopic Imaging of Micro/Nanocellulose Fibers and Fractured Sheets

SEM imaging was done for samples refined using both the LVB and SMC. For optimal imaging, a small quantity of diluted micro/nanocellulose slurry sample was evenly spread onto a glass slide and allowed to air-dry for 24 h at RT, ensuring individual fiber separation. Following drying, the samples were coated with a thin layer of gold (10 nm) via sputtering to avoid charging. SEM was used to measure fiber diameters, as shown in Figure 2. These methods facilitated detailed morphological analysis of fibers postrefining. Similarly, SEM was done for fractured tensile samples of micro/nanocellulose-filled epoxy sheets. After tensile testing, the sheets were kept in a zip-lock bag. Prior to SEM imaging, samples were cut to 20 mm in length from the fractured end and attached to the SEM stub so that imaging could be done at angles of 45 and 90 degrees. Optical microscopy (OM) was done for fractured tensile samples of micro/nanocellulose sheets and micro/nanocellulose-filled epoxy sheets conditioned at 98% RH.

### 2.7 Thermal Properties and Contact Angle of Micro/Nanocellulose-Filled Epoxy Sheets

Around 2–3 mg of pure micro/nanocellulose fibers, pure epoxy, and micro/nanocellulose fiber-filled epoxy material were loaded in a platinum pan, and thermogravimetric analysis (TGA) was carried out under a nitrogen atmosphere (flow rate of 100 mL/min). TGAs of all three categories of samples were carried out between 30 °C and 600 °C with a ramp rate of 10 °C/min. After TGA thermograms were analyzed, the residual weight loss (%) vs temperature was recorded.

The thermal conductivity (TC) of the epoxy and micro/nanocellulose fiber-filled epoxy materials was measured by using a TPS 500S hot disk sensor, as shown in Figure 6. The neat epoxy, which is isotropic, was subjected to TC measurement on one side only (surface of the specimen). In the case of micro/nanocellulose-filled epoxy, wherein fibers are oriented in a random orientation, TC was measured on both sides (both surfaces of the specimen). For both cases, a hot disk sensor of 2 mm diameter was used. As per the standard and hot disk sensor diameter, the specimen sizes of 20 × 20 × 2 mm^3^ and 15 × 20 × 1.5–2 mm^3^ were cut, respectively, for epoxy blocks and micro/nanocellulose-filled epoxy sheets using a cutting machine. All sample surfaces were smoothed and flattened to ensure good contact between the sample and the sensor. The sensor is placed between two identical pieces of the sample (sandwich pattern setup to ensure accurate measurements). The analyzer applies a heat pulse (heat input of 30–40 mW in the case of micro/nanocellulose-filled epoxy sheets and 100 mW in the case of pure epoxy) through the sensor and measures the temperature response to calculate TC. It should be noted that the hot disk sensor is a thin disk that acts as both a heat source and a temperature sensor. A current is passed through the sensor (time of 2.5 s, 29 °C in the case of filled epoxy and 5 s, 25 °C in the case of pure epoxy), causing it to heat up and allowing the sensor to measure the time-dependent temperature increase.

**Figure 6:**
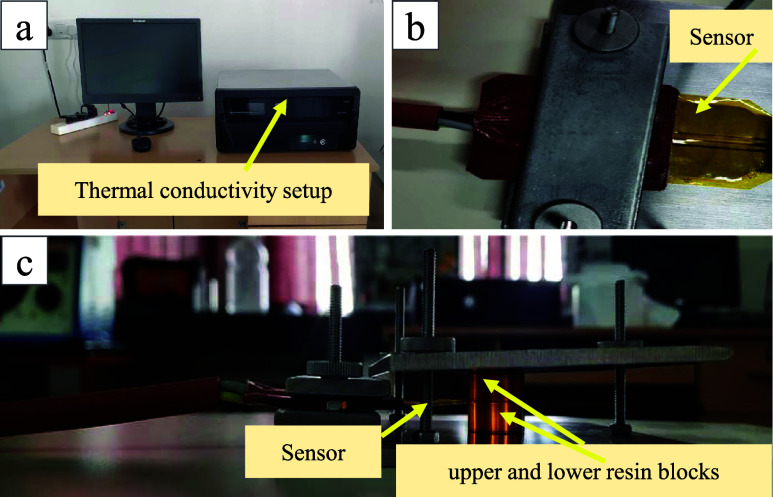
Thermal conductivity test setup. (a, b) Sensor and (c) sensor placed between two epoxy specimens (upper and lower resin blocks).

The surface hydrophobicity of the micro/nanocellulose fiber-filled epoxy sheets was measured by using a goniometer equipped with microscopy, which gives the contact angle of the system. A sessile drop method was considered, wherein a water droplet of 10 μL was deposited on the surface at a drop rate of 2 μL/s at 27 °C. All of the experiments were repeated 4–5 times to obtain the average value.

## 3 Results and Discussion

### 3.1 Microscopic Analysis of Micro/Nanocellulose Fibers

Fiber diameter and fiber length are reduced with a decrease in clearance from 0.1 to 0, as shown in Table 1. This uneven decrease also increased the aspect ratio by 10-fold. In the case of LVB refining, the fiber diameter is around 25 μm, and the aspect ratio is around 20. But in the case of (Table 1) SMC_0.1_60, the diameter is reduced to around 6 μm, and the aspect ratio is increased to 236. Further reduction in clearance (SMC_0_60 min) decreased the diameter to 330 nm and increased the aspect ratio of the fibers to 2353. For clarity in fiber diameters, SMC_0.4_60 min processed fibers were viewed under SEM, and the results are shown in Figure 2a,b. It can be noticed that individual fiber diameters are between 2.4 and 11.4 μm. At SMC_0_60 min clearance, it is important to note that more nanofibrils are formed, as shown in Figure 2c,d. Twisted and kinked fibers, along with fibers with split cell walls, are visible, but the number of fibrils is low because of low refining intensity (at a higher clearance of 0.4); hence, grinding at zero clearance for 60 min (SMC_0_60 min) was performed.

### 3.2 Tensile Testing of Micro/Nanocellulose Sheets (Subzero and Elevated Temperatures)

Due to the difficulty in handling and fixing these nonwoven sheets, tests were carried out at −5 °C, RT (23 °C), and 65 °C, and the corresponding stress–strain plots were recorded. The tensile properties of micro/nanocellulose sheets of LVB_0_20 min, SMC_0.1_60 min, and SMC_0_60 min are shown in Table 2 and Figures S1–S3. Table 2 shows the average values of the modulus of elasticity, tensile strength, and “work-to-fracture” of sheets measured at three different temperatures. The tensile modulus of sheets is high at subzero temperature (−5 °C) compared to elevated (65 °C) and room temperatures (23 °C) due to the brittle nature of LVB_20 min and SMC_0_60 min sheets. The tensile strength of sheets is high at elevated temperature (65 °C) compared to the other two temperatures due to the less moisture present in the sheets. SMC_0.1_60 sheet has low tensile properties (8–10 times if work-to-fracture is considered, 5–8 times if modulus and strength are considered) compared to SMC_0_60 min and LVB_0_60 min due to a high porous network. It is important to mention that LVB_20 sheets have a less porous network due to the presence of fines, and SMC_0_60 has a less porous network due to the presence of high aspect ratio fibers and micro/nanocellulose fibrils.

**Table 2:** Tensile Properties of Micro/Nanocellulose Sheets at Different Temperatures (−5 °C, RT, 65 °C)

cellulose nonwoven sheet	modulus of elasticity (GPa)			tensile strength (MPa)			work-to-fracture (J/m^3^) × 10^4^		
temperature	–5 °C	RT (23 °C)	65 °C	–5 °C	RT (23 °C)	65 °C	–5 °C	RT (23 °C)	65 °C
LVB_0_20 min	0.28 ± 0.1	0.2 ± 0.03	0.13 ± 0.01	3.6 ± 0.9	5.3 ± 0.3	6.0 ± 0.6	41 ± 5	46 ± 8	38 ± 7
SMC_0.1_60 min	0.01 ± 0.1	0.04 ± 0.02	0.02 ± 0.01	0.7 ± 0.3	1.2 ± 0.22	1.1 ± 0.04	3 ± 2	5 ± 1	5 ± 1
SMC_0_60 min	0.32 ± 0.1	0.3 ± 0.04	0.16 ± 0.02	7.3 ± 0.5	7.2 ± 0.31	9.6 ± 0.5	46 ± 11	38 ± 3	55 ± 12

SMC_0_60 min sheet has high tensile strength, E-modulus, and work-to-fracture at −5 °C, RT (23 °C), and 65 °C compared to LVB_0_20 min sheet and SMC_0.1_60 sheets, due to the presence of nanocellulose fibrils (Figure 2c,d). LVB_0_20 min sheet has high failure strain and work-to-fracture at RT (23 °C) due to a greater number of fines, which have lengths below 2 μm. At 65 °C, the SMC_0_60 min sheet has high work-to-fracture due to the loosening of the high aspect ratio fiber network and also due to the high tensile strength compared to other sheets.

At room temperature (shown in Figure S1), in the case of the SMC_0_60 min sheet, the elastic region lies between 0 and 1%, the plastic region lies between 1 and 7%, and the fracture region lies between 7 and 7.5%. In the case of the SMC_0.1_60 min sheet, the elastic region lies between 0 and 0.2%, the plastic region lies between 0.2 and 5%, and the fracture region lies between 5 and 6.2%; hence, the lowest work-to-fracture. In the case of the LVB_0_20 min sheet, the elastic region lies between 0 and 0.5%, the plastic region lies between 0.5 and 9%, and the fracture region lies between 9 and 10%; hence, the highest work-to-fracture. At −5 °C (see Figure S2), in the case of the SMC_0_60 min sheet, the elastic region lies between 0 and 1.5%, and the fracture region lies between 9 and 10.5%. In the case of the SMC_0.1_60 min sheet, the elastic region lies between 0 and 2%, and the fracture region lies between 5.2 and 6.2%. In the case of the LVB_0_20 min sheet, the elastic region lies between 0 and 1%, and the fracture region lies between 4.5 and 5.5%. At 65 °C (See Figure S3), in the case of the SMC_0_60 min sheet, the fracture region lies between 9.5 and 11%. In the case of the SMC_0.1_60 min sheet, the fracture region lies between 7.5 and 8.5%. In the case of the LVB_0_20 min sheet, the fracture region lies between 9.5 and 10.5%.

### 3.3 Tensile Testing of Epoxy Specimens and Micro/Nanocellulose-Filled Epoxy Sheets

These sheets can be used in packaging and other exterior applications; hence, tensile tests were carried out at −20 °C, RT (23 °C), and 85 °C, and the corresponding stress–strain plots were recorded. The tensile test results of pure epoxy specimens at all three temperatures (Figure 7a), pure micro/nanocellulose sheets (Figure 7b), and micro/nanocellulose-filled epoxy sheets at RT (23 °C) are shown in Figure 8a. Unlike fiber-reinforced epoxy composites, epoxy resin (65 MPa) has an 8-fold higher tensile strength than a pure SMC_0_60 sheet (8 MPa). Hence, the micro/nanocellulose-filled epoxy sheet strength is reduced to 30 MPa, and it is in alignment with the simple rule of mixture for discontinuous and randomly oriented fiber-based composites. Due to porous nonwoven sheets and shorter lengths, these sheets are inferior to epoxy resins in terms of mechanical properties, despite having excellent bonding with epoxy resins. As mentioned earlier, resin contents are very high (55–65 wt %), and sheets are not subjected to any additional pressure; hence, strength and modulus values are low. From Figure 7a, it can be noticed that epoxy resin became brittle at −20 °C and became softer at 80 °C due to the shrinkage and expansion of the 3D-networked polymer, respectively, which could be due to the amorphous nature of the epoxy resins. Table 3 shows the average values of tensile stress, tensile modulus, and strain for the micro/nanocellulose-filled epoxy sheets and pure epoxy dog bone specimens at all three temperatures. Figure 8a–c shows the tensile stress–strain diagrams of micro/nanocellulose-filled epoxy sheets at three different temperatures.

**Figure 7:**
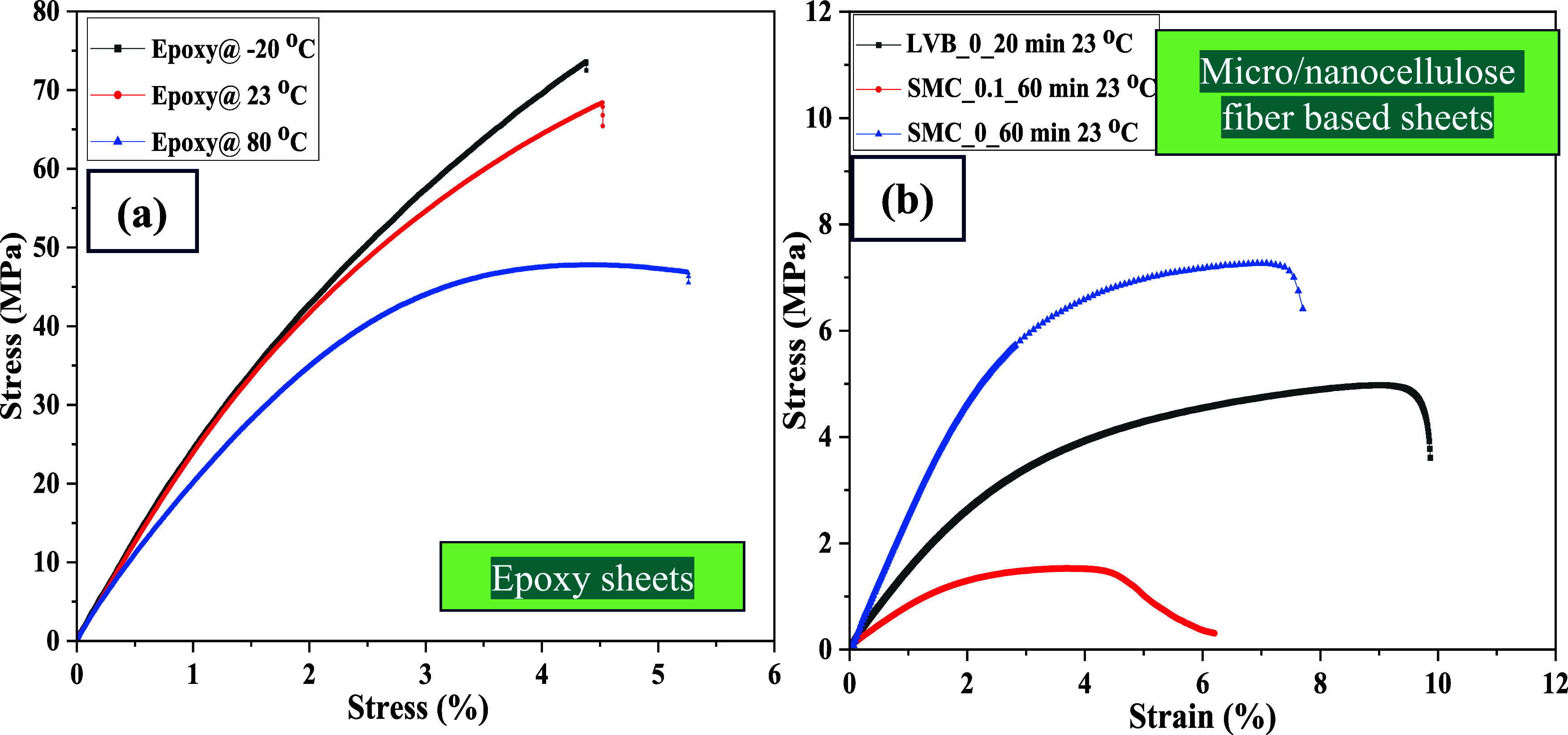
(a) Tensile stress–strain curves of epoxy matrix sheets tested at different temperatures of −20 °C, 23 °C, 80 °C; (b) micro/nanocellulose-based nonwoven sheets (without epoxy) tested at 23 °C.

**Figure 8:**
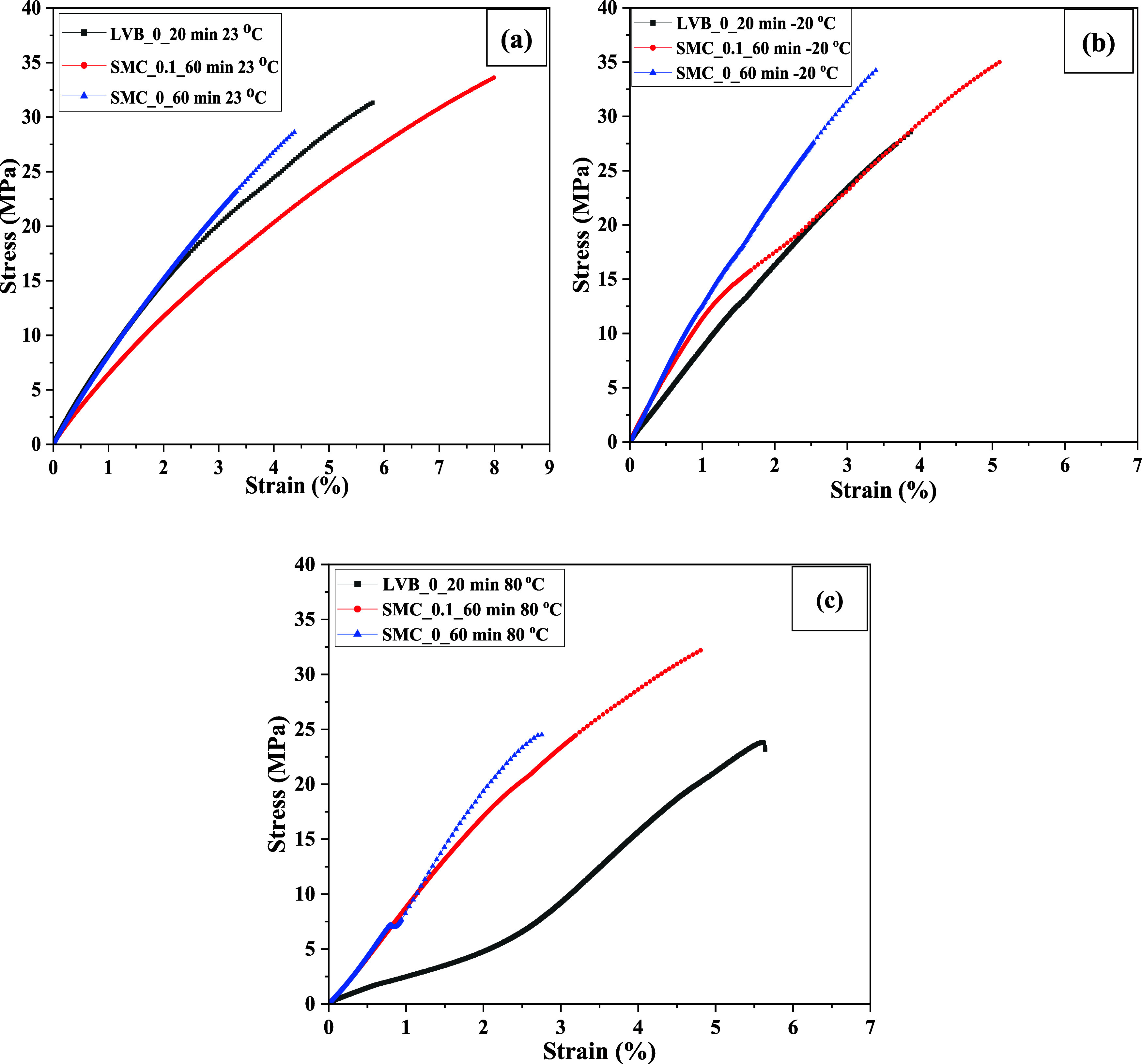
(a) Tensile stress–strain diagram of micro/nanocellulose-filled epoxy sheets tested at 23 °C, (b) −20 °C, and (c) 80 °C.

**Table 3:** Tensile Properties of Micro/Nanocellulose-Filled Epoxy Sheets at Different Temperatures (−20 °C, RT, 80 °C)

cellulose epoxy sheets	modulus of elasticity (GPa)			tensile strength (MPa)			work-to-fracture (J/m^3^) × 10^4^		
temperature	–20 °C	RT (23 °C)	80 °C	–20 °C	RT (23 °C)	80 °C	–20 °C	RT (23 °C)	80 °C
LVB_0_20 min_epoxy sheet	1.3 ± 0.4	0.9 ± 0.02	0.7 ± 0.1	29 ± 1.4	33 ± 1.5	26 ± 1.3	80 ± 20	200 ± 40	223 ± 71
SMC_0.1_60 min_epoxy sheet	1.2 ± 0.2	0.7 ± 0.1	0.9 ± 0.01	36 ± 1.4	32 ± 1.6	33 ± 1	155 ± 13	308 ± 12	250 ± 18
SMC_0_60 min_epoxy sheet	1.4 ± 0.2	1 ± 0.2	1 ± 0.1	35 ± 1.0	30 ± 1.2	28 ± 4	107 ± 11	139 ± 36	89 ± 42
Epoxy	2.7 ± 0.1	2.4 ± 0.1	1.8 ± 0.3	74 ± 11	66 ± 8.8	48 ± 4	188 ± 16	189 ± 10	185 ± 19

One of the authors reported earlier that the tensile strength (70–20 MPa) and modulus (2.5–0.75 GPa) of neat resin showed a nonlinear decrease from −20 to 150 °C and also clarified that the brittle nature of resin resulted in cleavage fracture at −20 °C.^25^ Similar to these results, tensile strength and modulus of micro/nanocellulose-filled epoxy sheets are high at subzero temperature (−20 °C) compared to elevated temperatures (80 °C) and room temperature (23 °C) due to the shrinkage and restriction of chain movements of polymer material. As expected, the failure strain and work-to-fracture of micro/nanocellulose-filled epoxy sheets are low at subzero temperature (−20 °C—Figure 8b) compared to elevated (80 °C–Figure[Fig fig8]c) and room temperature (23 °C), which need to be considered in the design of components.

It should be emphasized that liquid epoxy plays an important role in the mechanical properties at different temperatures. Work-to-fracture of LVB_20_epoxy sheets at – 20 °C is 80 × 10^4^ J/m^3^, but the value is increased by almost 3-fold with the increase in temperature to 80 °C, which is due to the increase in failure strain from 3.3% to 5.3%. Similarly, the value is increased by almost 1-fold in the case of SMC_0.1_60_epoxy sheet, but no change was found in the case of SMC_0_60_epoxy and pure epoxy sheets. Due to excellent bonding between the nanofibrils of the SMC_0_60 sheet and epoxy, the whole sheet became nonporous, and there was no scope for stretching and sliding during tensile testing. The SMC_0.1_60_epoxy sheet, having a more porous network (due to lack of fines and nanofibrils), is found to have the highest work-to-fracture at all temperatures, as shown in Table 3, because resin content was very high (65 wt %).

The modulus of elasticity is low for the SMC_0.1_60_epoxy sheet at −20 °C compared to the SMC_0_60_epoxy sheet and LVB_0_20_epoxy sheet but high compared to the respective RT and 80 °C data, and this could be due to the porosity that exists as explained earlier. The modulus of elasticity is high for SMC_0_60_epoxy sheet at −20 °C, RT, 80 °C, compared to the other two sheets, which could be due to the low porosity, high aspect ratio fibers (Table 1), and high fibrillation (Figure 2c,d). At subzero (−20 °C) and elevated (80 °C) temperatures, the high tensile strength is observed for the SMC_0.1_60_epoxy sheet, followed by the SMC_0_60_epoxy sheet and LVB_0_20_epoxy sheet due to excess resin content. At −20 and 80 °C, the SMC_0_60_epoxy sheet is found to have low work-to-fracture, which could be due to transverse microcracks (delamination between layers) existing in this sheet, which have less impact on modulus but high impact on strength, because propagation of microcracks is clearly visible (Figure 9).

**Figure 9:**
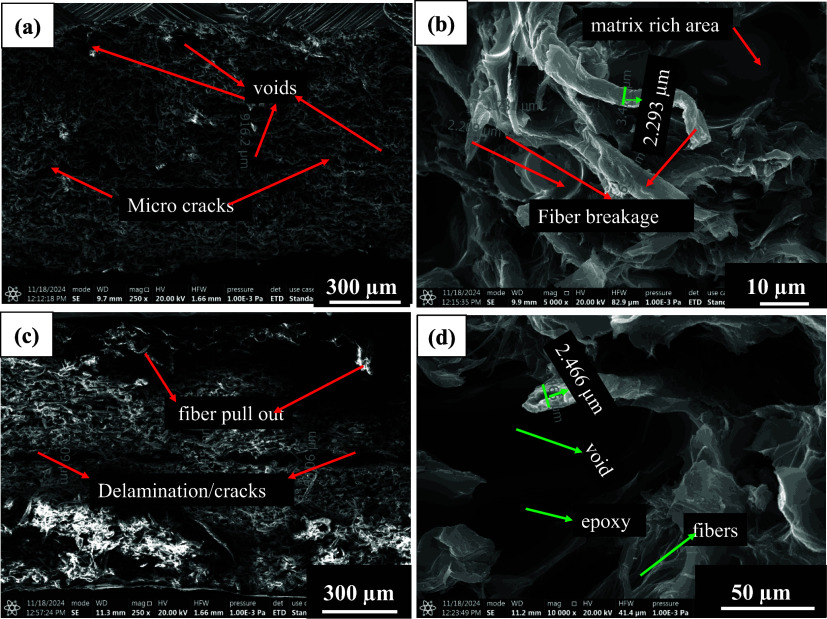
SEM tensile fractography of SMC_0_60 min_epoxy (a, b −45°), (c, d – 0°) sheet tested at −20 °C.

Failure strain is high at RT, followed by 80 °C, and −20 °C for all filled epoxy sheets. At −20 °C, the high failure strain was observed for the SMC_0.1_60 min sheet, followed by SMC_0_60 min and LVB_0_20 min, which could be due to the high porous network of fibers. At RT, high failure strain was observed for SMC_0.1_60 min, followed by LVB_0_60 and SMC_0_60 min. At 80 °C, the LVB_0_20 min micro/nanocellulose-filled epoxy sheet has a high failure strain, followed by SMC_0.1_60 min and SMC_0_60 min, which could be due to the loss of moisture and softening of lignin matrix. SMC_0.1_60_epoxy is taking high work up to fracture at RT and 80 °C, followed by LVB_0_20 min and SMC_0_60_epoxy sheet. At subzero temperature, SMC_0.1_60 min takes more work up to fracture, followed by SMC_0_60 min and LVB_0_20 min.

To understand the influence of relative humidity (RH) on tensile properties, three sheets (LVB_0_20_epoxy, SMC_0.1_60_epoxy, and SMC_0_60_epoxy) were tested at 98% RH, and properties were compared, as shown in Figure 10. It is clear that the RH has decreased the strength and stiffness of filled epoxy sheets by 30–50%, and a significant reduction was found in the case of SMC_0_60_epoxy sheets (27 to 17 MPa), which could be due to more number of nanocellulose fibrils. Elongation at break and work-to-fracture (not calculated) also reduced, which could be due to moisture absorption and subsequent swelling of sheets.

**Figure 10:**
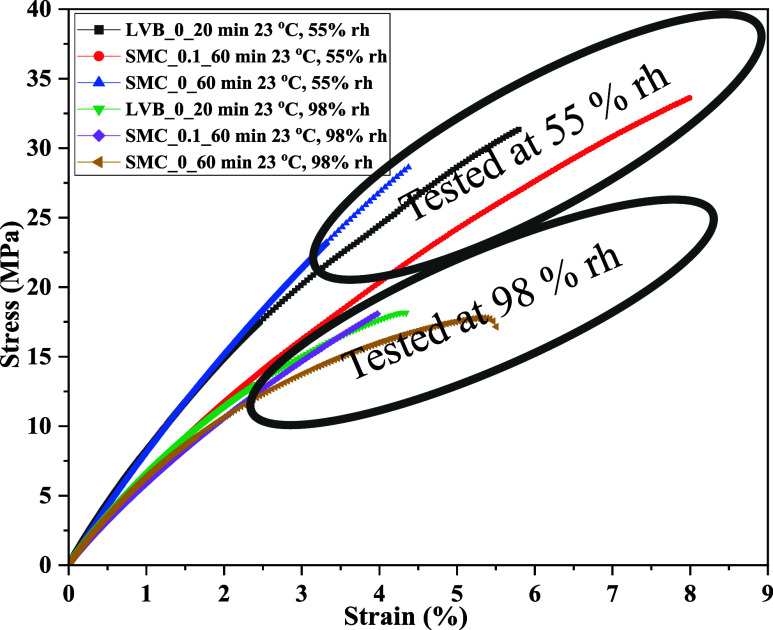
Tensile stress–strain diagram of micro/nanocellulose-filled epoxy sheets at different relative humidities (RH) of 55% and 98%.

### 3.4 Tensile Fractography of Micro/Nanocellulose Sheets and Micro/Nanocellulose-Filled Epoxy Sheets

OM was used to study the fractography of micro/nanocellulose sheets. Figure 11 shows the fractography of sheets tested at −5 °C, and Figure 12 shows the fractography of micro/nanocellulose-filled epoxy sheets tested at 98% RH. Ductile fracture was noticed in the case of sheets, but the level of ductility was higher in the case of the SMC_0_60 min sheet, as evidenced by the high fracture angle (31.5 degrees). Due to the nonwoven sheet formation, fibers are interlinked to each other, and tensile fracture is always found to be ductile, as shown in Figure 11.

**Figure 11:**
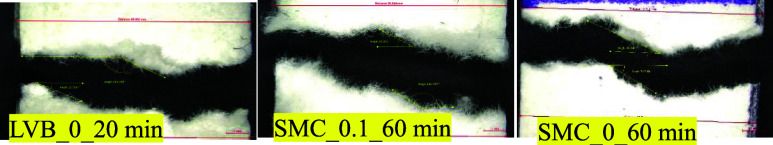
OM tensile fractography of LVB_0_20 min, SMC_0.1_60 min, and SMC_0_60 min sheets tested at −5 °C.

**Figure 12:**
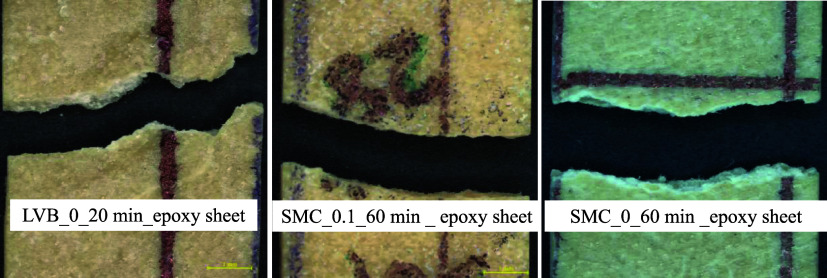
OM tensile fractography of micro/nanocellulose-filled epoxy sheets tested at 23 °C and 98% RH.

But the micro/nanocellulose-filled epoxy sheets (SMC_0_60 min_epoxy sheet) revealed slightly ductile fracture, as shown in Figure 12, due to the excellent bonding between cellulose nanofibrils and epoxy. Similarly, LVB_20 min_epoxy sheet also revealed a slightly ductile fracture due to the presence of fines, but a slightly brittle fracture (smooth fracture) was noticed in the case of SMC_0.1_60 min_epoxy sheet due to the absence of fines and nanofibrils and also due to the excess resin content. The interfacial failure between regenerated cellulose (lyocell) fiber and epoxy matrix was also found to be brittle in nature.^18^

SEM fractography (cross section) of micro/nanocellulose-filled epoxy samples is shown in Figures 9, 13, and 14. These filled epoxy sheets were tested at three different temperatures (−20 °C, RT, 80 °C). Fractography of RT tested micro/nanocellulose-filled epoxy sheets is shown in Figure S4 (LVB_20_epoxy), Figure S5 (SMC_0.1_60_epoxy), and Figure 13 (SMC_0_60_epoxy). From these three fractography images, it can be concluded that the fiber/fine diameter is larger in LVB_20_epoxy (6–8 μm), and a small amount of nanofibrils (200–500 nm) is also visible, which eventually leads to an increase in modulus and strength. In the case of SMC_0.1_epoxy sheets, the fiber diameter is reduced to 5 μm, but the packing density seems to be low, which can be witnessed in the form of microvoids. The bonding seems to be weak between cellulose and epoxy due to a smaller number of nanofibrils (155–400 nm). In the case of the SMC_0 sheet, voids are absent, the fiber diameter is reduced to 4 μm, and a huge amount of nanofibrils (85–300 nm) was found, which eventually increased the packing density. Due to the issues in laying sheets, bonding between two sheets resulted in a crack, as shown in Figure 13a, which could be the reason for the reduction in tensile strength.

**Figure 13:**
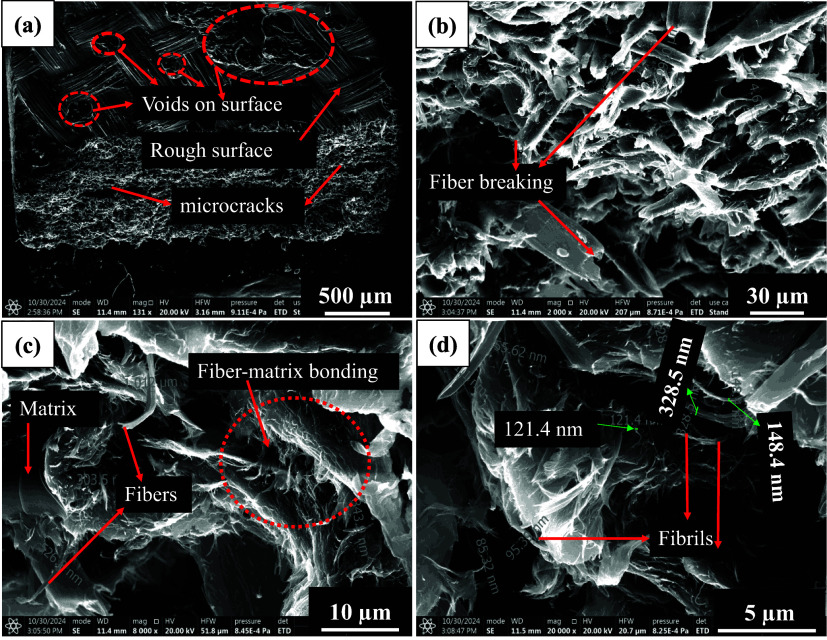
SEM tensile fractography of SMC_0_60 min_epoxy sheet tested at RT (23 °C). (a) Microcracks caused due to tensile load, (b) fiber breakage with less pullout, (c) regions of matrix and fiber–matrix interface, and (d) nanofibrils in the interfacial region.

**Figure 14:**
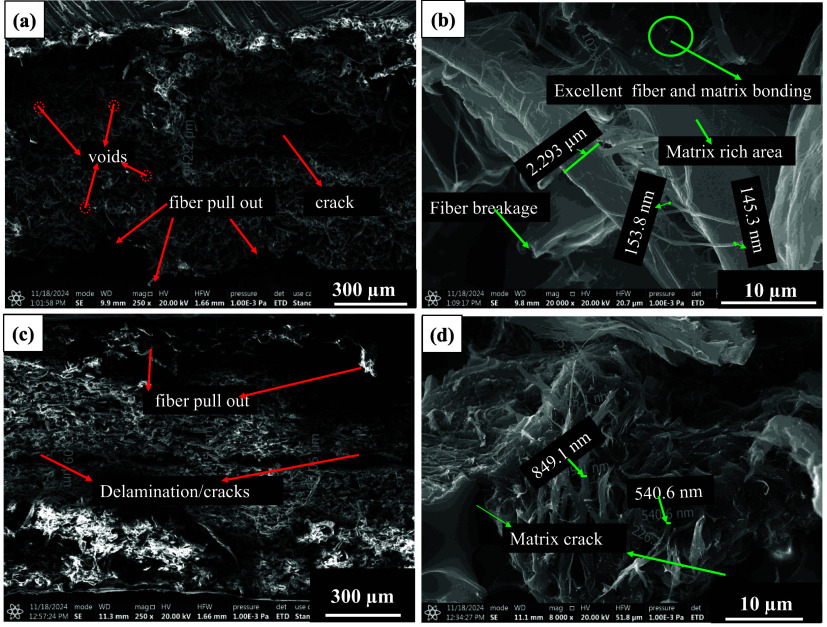
SEM tensile fractography of SMC_0_60 min micro/nanocellulose-filled epoxy sheet (a, b −45°)–(c, d −90°) tested at 80 °C.

Fractography of −20 °C tested micro/nanocellulose-filled epoxy sheets is shown in Figure S6 (LVB_20_epoxy), Figure S7 (SMC_0.1_60_epoxy), and Figure 7 (SMC_0_60_epoxy). As shown in Figure S6, it can be noticed that a few microvoids are present, and fiber diameters are between 4–10 μm. Due to the fines and shrinkage of the polymer chains, bonding is good, and fiber fracture resembles a brittle fracture of the epoxy, leaving no fiber pullout in the cross-section morphology. As shown in Figure S7, void diameters are between 32 and 38 μm, and fiber diameters are between 7 and 15 μm. Due to subzero temperatures, partial fiber pullout was visible, and fiber splitting (1.8 μm width) was noticed in the cross-section morphology. As shown in Figure 14, excellent bonding is visible due to nanofibrils present in the SMC_0_60 min_epoxy sheet and the low diameter of fibers (2–4 μm). Due to the shrinkage of molecules, a slight pullout was observed in the cross-section morphology of micro/nanocellulose-filled epoxy sheets.

Fractography of 80 °C tested filled epoxy sheets is shown in Figure S8 (LVB_20_epoxy), Figure S9 (SMC_0.1_60_epoxy), and Figure 14 (SMC_0_60_epoxy). LVB_20_epoxy sheets revealed voids, which could be due to the softening of the interface, and individual fiber diameters are between 3 and 9 μm. Though nanofibrils (226–840 nm) and fiber splits (200–1200 nm width) are present, bonding seems to be weaker, which eventually resulted in lower strength and modulus.

SMC_0.1_60_epoxy sheets revealed voids, which could be due to the pullout of the fibers at elevated temperature, and individual fiber diameters are between 3 and 9 μm. Though nanofibrils (400–800 nm) are present, bonding seems to be weaker due to the absence of fines and a smaller number of nanofibrils (as shown in Figure S9), which led to the lowest strength and modulus. SMC_0_60_epoxy sheets revealed no voids and a greater number of nanofibrils (230–500 nm, as shown in Figure 14), which could be due to the extensive refining and excellent bonding with the epoxy matrix, which altogether increased the strength and modulus of SMC_0_60_epoxy sheets.

### 3.5 TGA and Thermal Conductivity of Micro/Nanocellulose-Filled Epoxy Sheets

TGA results revealed better thermal stability for micro/nanocellulose-filled epoxy sheets compared to pure epoxy or nanocellulose, as shown in Table S1 (considering 220 °C). The TGA curves (Figure 15) reveal that the onset of major thermal degradation for the micro/nanocellulose-filled epoxy sheets occurs between 220 and 230 °C, whereas the neat epoxy begins to degrade at approximately 200 °C. The slight increase in degradation onset temperature and higher residual char yield observed in the micro/nanocellulose-filled epoxy sheets indicate enhanced thermal stability due to the micro/nanocellulose sheets. The same is true considering 430 °C, wherein epoxy weight loss is around 69%, and nanocellulose weight loss is around 80%, but micro/nanocellulose-filled epoxy sheets revealed weight loss between 55 and 60%. Due to the more porous network, SMC_0.1_60_epoxy seems to be thermally stable compared to the other two sheets. Weight loss was found to be higher for micro/nanocellulose-filled epoxy sheets between 430 and 600 °C due to the composite nature of the sheet, which indicates that these cellulose-filled epoxy sheets can be used only up to 220 °C. From Figure 15, it can also be noticed that the degradation of micro/nanocellulose-filled epoxy sheets is slow and gradual compared to pure epoxy or pure micro/nanocellulose sheets.

**Figure 15:**
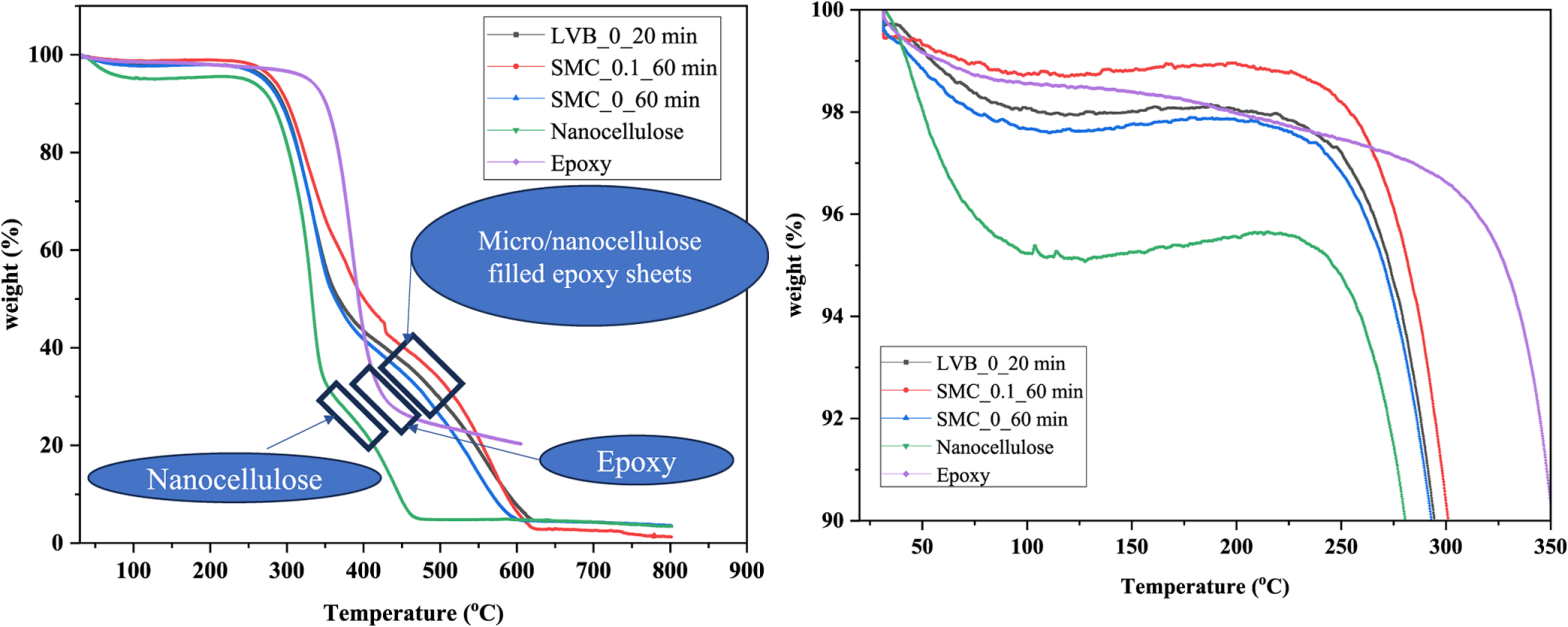
Thermogravimetric analysis (TGA) of micro/nanocellulose sheet, pure epoxy, and micro/nanocellulose-filled epoxy sheets.

From Table 4, it can be noticed that the thermal conductivity (TC) of filled epoxy sheets is high compared to pure epoxy, which could be due to the presence of partially crystalline micro/nanocellulose fibers. Though fibers are randomly oriented, crystallinity is slightly higher due to lengthy fibers (oriented in one direction but shorter lengths) and their bonding with the epoxy matrix. Bottom surface is rich in epoxy due to the contact with flat mold; hence, lower TC values are observed because of the smooth surface, whereas the top surface is rich in cellulose sheet; hence, uneven surface texture is noticed, which led to an increase in TC value. If the sheet’s top surface is considered, the TC value of SMC_epoxy sheets is almost 72–77% higher than epoxy block. Similarly, the LVB_epoxy sheet also revealed 50% higher TC value compared to epoxy due to the presence of a greater number of short-length fibers (fines, etc.). The density of the filled epoxy sheets is also presented in Table 4, and pure epoxy is slightly denser than the filled epoxy sheets. SMC_0.1_60_epoxy sheets revealed a slightly higher density than the other two sheets, with high porosity and high resin content. These epoxy-filled sheets can be explored in thermal packaging due to low density, high thermal conductivity, and better thermal stability, as reported in the TGA thermogram (Figure 15). From a practical standpoint, these results suggest that the developed micro/nanocellulose-filled epoxy sheets can safely withstand temperatures up to ≈220 °C without significant mass loss, making them suitable for moderate-heat packaging (food), insulation panels, and electronic casing applications, where dimensional stability and partial biodegradability are desirable. Above this temperature range, progressive decomposition of both the cellulose and epoxy phases occurs. Therefore, while these micro/nanocellulose-filled epoxy sheets are not intended for high-temperature structural use, their stability within this range supports potential applications in thermal insulation and eco-friendly packaging materials.

**Table 4:** Physical and Thermal Properties of Micro/Nanocellulose-Filled Epoxy Sheets

			thermal conductivity (W/m. K)		TGA analysis
S. No	material	density (ρ −g/cm^3^)	top surface	bottom surface	cumulative weight loss (%) up to 430 °C
1	LVB_0_20 min_epoxy sheet	0.953 ± 0.01	0.33 ± 0.01	0.29 ± 0.02	58.23
2	SMC_0.1_60 min_epoxy sheet	0.972 ± 0.01	0.39 ± 0.01	0.34 ± 0.01	55.99
3	SMC_0_60 min_epoxy sheet	0.969 ± 0.01	0.38 ± 0.01	0.32 ± 0.01	60.17
4	Epoxy block	1.16 ± 0.01	0.22 ± 0.02		68.73

### 3.6 Contact Angle of Micro/Nanocellulose-Filled Epoxy Sheets

Contact angle measurements of top (rough) and bottom (smooth) surfaces of micro/nanocellulose-filled epoxy sheets are shown in Table 5. The top surface has a lower contact angle (66–72°), while the bottom surface has a higher contact angle (96–118°). This variation can be attributed to the processing conditions and the resulting surface morphology. During vacuum-assisted fabrication, the upper surface is more exposed to air, resulting in uneven curing and localized resin depletion, which can result in microvoids and rougher regions (as shown in Figure S5a). The images clearly highlight the microvoids and roughness variations responsible for the lower contact angles on the top surface (as shown in Figure S5a). Although roughness and wettability are two distinct surface properties, surface wettability can be controlled by incorporating microstructures that introduce roughness, so roughness is typically studied in conjunction with surface contact angle measurements.^26^ These microvoids trap air and increase surface wettability, resulting in a smaller apparent contact angle. The top surface thus behaves as a partially porous and hydrophilic interface, influenced by the presence of exposed cellulose fibrils and insufficient resin coverage/depletion. In contrast, the bottom surface of the sheets, which remained in contact with the smooth mold surface while curing, has a denser and more uniform epoxy layer with fewer voids. This results in increased surface smoothness and resin continuity, reducing water spreading and producing a more hydrophobic surface (118°), as shown in Figure S10. Surface asymmetry is common in vacuum bagging or compression-molded epoxy composites, where differential resin migration and pressure distribution during curing influence the final topography.^27^

**Table 5:** Contact Angle Measurements of Micro/Nanocellulose-Filled Epoxy Sheets

S.No	sheets	contact angle (°)—top surface	contact angle (°)—bottom surface
1	LVB_0_20 min_epoxy sheet	66.5 ± 8.4	118 ± 1
2	SMC_0.1_60 min_epoxy sheet	71 ± 4.3	96 ± 1.4
4	SMC_0_60 min_epoxy sheet	66 ± 5.6	110 ± 4.5

SMC_0.1_60_epoxy has slightly different contact angle values than those of LVB_0_20_epoxy and SMC_0_60_epoxy. This variation can be attributed to differences in the fiber size distribution and fibrillation intensity. The SMC_0.1_60 sample had fewer fines and nanofibrils, and more voids (as shown in Figure 3c,d), resulting in heterogeneous dispersion and potentially resin-rich domains that disrupt uniform wetting behavior (Table S2). Furthermore, the microvoid content was higher in this sheet, as seen during SEM analysis (Figure S5a), which contributed to fluctuations in the measured contact angle. The top surface’s moderate hydrophilicity (71°) suggests that fewer hydroxyl groups from cellulose are available at the interface due to partial resin encapsulation. This variation in hydrophobicity can have important implications for packaging applications. The more hydrophobic surface would act as a moisture barrier, preventing water penetration and protecting packaged goods from humidity. Meanwhile, the moderately hydrophilic surface can facilitate adhesion to other materials (films, foils, or coatings) during multilayer packaging fabrication. Such dual-surface behavior offers design flexibility—enabling one side of the sheet to serve as a barrier layer while the opposite side ensures interfacial bonding in lamination or structural assembly. The contact angles measured (as shown in Table 5) were between 66° (hydrophilic) and 118° (hydrophobic), which are useful for moisture-resistant packaging and coatings. The combination of moderate hydrophobicity and good thermal conductivity (Table 4) implies that these filled epoxy sheets can be effectively used in biodegradable/sustainable packaging laminates and barrier layers, where moisture control is crucial.

### 3.7 Applications in Food Packaging

Since these micro/nanocellulose-filled epoxy sheets are partially biodegradable, have optimum strength and stiffness, and have better thermal stability, these sheets are recommended for food packaging. Beyond aesthetics, food packaging materials are responsible for protecting food from contamination and damage from handling/transportation, preserving freshness, extending shelf life, and providing information about the food to the supply chain for efficient inventory management and to the consumers for making the right purchase decisions.^28^ Traditionally, materials such as glass, metals (aluminum, tin, and steel), paper and paper boards, and plastics were used for packaging. However, modern innovations in plastic technology have replaced other materials with plastics due to their lower cost, improved functionality, tunability (rigid to flexible), durability, and lightweight. According to Plast India, the Indian packaging industry uses around 59% of all plastics produced in India.^29^ Often, plastics fall under two categories: thermoplastics and thermosets.^30,31^ Thermosets are strong and durable compared to thermoplastics and are often used in packaging applications, where durability and heat resistance are essential. Thermosets comprise around 20% of all polymeric materials produced today.^32^

Plastic pollution is a growing concern. Environmental awareness and sustainable living concepts have directed the United Nations to propose sustainable development goals (SDGs) as guidelines for achieving long-term sustainability and reducing environmental distress.^33^ Significant interests lie in developing biodegradable alternatives to thermosets to align with the SDGs. One approach toward sustainability would be to incorporate biodegradable materials such as cellulose in a traditional thermoset matrix intelligently.^34^ Including biodegradable materials would reduce the overall weight percentage of nonbiodegradable polymers in the final product and improve the functional properties of thermosets. Thus, these micro/nanocellulose-filled epoxy sheets will have both nonbiodegradable (epoxy) and biodegradable (micro/nanocellulose) components, which would help achieve fast partial degradability and fragmentation of the polymer matrix, which may further accelerate its degradation (Figure S11).

The tensile properties presented in Table 3 and the TGA data shown in Figure 15 indicate that micro/nanocellulose-filled epoxy sheets retain most of their mechanical properties at elevated temperatures and exhibit high thermal stability. It is well established that thermosets are highly resistant to chemical attack due to their highly cross-linked molecular structure, which prevents chemicals from penetrating and reacting with the material. These properties make them suitable for demanding applications where high-temperature strength is required, such as components in ovens, chain guides, and nozzles for cleaning equipment. The filled epoxy sheet material also exhibits low thermal conductivity (Table 4). The Epofine 1555 resin used in this work shows low flammability.^35^ These properties further enhance the suitability of filled epoxy sheets for food processing environments. The material shows low water absorptivity (ranging from 1.6 to 4.5% for three micro/nanocellulose-filled epoxy sheets) at 60 °C and 98% relative humidity (Table S2). It was observed that SMC_0.1_60 min sheets have low water absorptivity (Table S2) due to a high epoxy content. Such low water absorption suggests that the material will have excellent water vapor barrier properties.^36^ Food handling materials must possess low water permeability, as this is crucial for preserving product quality, ensuring safety, and extending shelf life.^37^ Given this reliance, the micro/nanocellulose epoxy-filled sheets developed in this work will be a suitable, eco-friendly alternative to conventional plastics currently used in the food industry.

## 4 Conclusions

Bleached pulp was subjected to refining using a Lab Valley beater (LVB) and Super masscolloider (SMC), and the resulting porous nonwoven sheets are categorized as LVB_0_20, SMC_0.1_60, and SMC_0_60. These nonwoven sheets were found to have micro/nanocellulose fibers (all three sheets) and nanocellulose fibrils (SMC_0_60 sheet). Later, these nonwoven sheets were mixed with liquid epoxy matrix using the hand layup technique, and finally, solid sheets were made using the vacuum bagging technique, followed by curing at 160 °C for 3 h. Tensile properties of pure nonwoven sheets and filled epoxy sheets were measured at subzero, RT, and elevated temperatures. The SMC_0_60 sheet (without epoxy) revealed 6–10-fold higher tensile strength and modulus at RT, subzero, and elevated temperatures compared to the SMC_0.1_60 sheet, which could be due to the presence of more nanocellulose fibrils leading to a smaller number of microvoids in the sheet. When data were compared among micro/nanocellulose-filled epoxy sheets, surprisingly, the work-to-fracture of the SMC_0.1_60_epoxy sheet was 2-fold higher than the SMC_0_60_epoxy sheet and the pure epoxy sheet at RT and 80 °C, which could be due to the high elongation, high resin content, and a smaller number of nanocellulose fibrils existing in the SMC_0.1_60_epoxy sheet. At −20 °C, both SMC_epoxy sheets revealed higher tensile strength than LVB_epoxy sheets, which could be due to the high cellulose content found in SMC refined sheets. At all three temperatures, epoxy resin strength and modulus are almost 2-fold higher than those of filled epoxy sheets, which could be due to the nonwoven sheets and high porosity in the sheets.

The micro/nanocellulose sheets used in this study primarily act as biofillers rather than mechanical reinforcements. The incorporation of these fillers is intended to improve the thermal stability, partial biodegradability, and moderate mechanical performance. The observed decrease in tensile strength and modulus is attributed to the physical characteristics of the nondensified fillers and the limited interfacial load transfer when compared to the pure epoxy sheet. The aim is to manufacture a sheet with high work-to-fracture, like the SMC_0.1_60_epoxy sheet, which is partially biodegradable and recommended for thermal packaging with a slight compromise on strength and stiffness.

SMC_epoxy sheets have a high tensile strength and modulus at −20 °C compared to RT and 80 °C due to increased shrinkage of epoxy resin. It is found that work-to-fracture of micro/nanocellulose-filled epoxy sheets decreased at −20 and 80 °C due to shrinkage and postcuring effect, respectively. But in the case of LVB, degradation of lignin at elevated temperatures also contributed to a reduction in work-to-fracture and modulus. However, the LVB sheet shows high tensile strength at high relative humidity due to the presence of high lignin content and a larger number of fines. This study offers insights into the development of low-density (0.96 g/cm^3^), high-thermal conductivity (0.38 W/m·K), better thermal stability (until 220 °C), partially biodegradable, high work-to-fracture micro/nanocellulose-filled epoxy sheets suitable for RT and elevated temperatures, but note the decrease in strength and modulus at these temperatures. By applying pressure (decreasing the resin content) and varying the refining intensity, different sheets can be made, which can be made available for different packing applications.

## Supplementary Material



## Data Availability

The data underlying this study are available in the published article and its Supporting Information.

## Notes

Figures S1–S11 and Tables S1–S2. Stress–strain plots of Micro/nanocellulose sheets at different temperatures of 23 °C (Figure S1), −5 °C (Figure S2), 65 °C (Figure S3), SEM images of tensile fractography of LVB_0_20 min (Figure S4), SMC_0.1_60 min (Figure S5) epoxy sheets at RT, SEM images of tensile fractography of LVB_0_20 min (Figure S6), SMC_0.1_60 min (Figure S7) epoxy sheets at −20 °C, SEM images of tensile fractography of LVB_0_20 min (Figure S8), SMC_0.1_60 min (Figure S9) epoxy sheets at 80 °C, contact angle measurements on top and bottom surfaces of micro/nanocellulose-filled epoxy sheets (Figure S10), stereomicroscopy image after 30 days of biodegradability on SMC sheet (Figure S11), thermogravimetric analysis of micro/nanocellulose-filled epoxy sheets, pure epoxy, and micro/nanocellulose sheets (Table S1), and water absorptivity on micro/nanocellulose-filled epoxy sheets (Table S2) (PDF)
