# Supplementary material for: Mechanical Properties of Micro/Nanocellulose-Filled Epoxy Sheets at Subzero and Elevated Temperatures
Source: ACS Omega. 2025 Dec 17;10(51):63102–16. doi: 10.1021/acsomega.5c09299 (PMC12756841; doi:10.1021/acsomega.5c09299)
Supplement: Supplementary file 1 [file ao5c09299_si_001.pdf]

Supporting Information

Mechanical Properties of Micro/Nanocellulose filled Epoxy Sheets at Subzero and Elevated Temperatures

Pallavi Gulipalli*[1], Chandra Babu Mallineni[1], Ramesh Adusumalli[1], Ramendra Kishor Pal[1]

[1]Department of Chemical Engineering, Birla Institute of Technology and Science, Pilani, Hyderabad Campus, Jawahar Nagar, Kapra Mandal, Medchal District, Telangana 500078, India

Table of Contents

1. **Fig. S1:** Tensile stress-strain curves of micro/nanocellulose sheets tested at 23 ºC
2. **Fig. S2:** Tensile stress-strain curves of micro/nanocellulose sheets tested at -5 ºC
3. **Fig. S3**: Tensile stress-strain curves of micro/nanocellulose sheets tested at 65 ºC
4. **Fig. S4**: SEM images of tensile fractography of LVB_0_20 min_ epoxy sheet tested at RT
5. **Fig. S5:** SEM images of tensile fractography of SMC_0.1_60 min_ epoxy sheet tested at RT (23 ºC)
6. **Fig. S6:** SEM images of tensile fractography of LVB_0_20 min_epoxy sheet (**a**, **b**, **c** - 45º) - (**d**, **e**, **f** – 90º) tested at -20 ºC
7. **Fig. S7:** SEM images of tensile fractography of SMC_0.1_60 min_epoxy sheet (**a**, **b**, **c** – 45º) - (**d**, **e**, **f** – 90º) tested at -20 ºC
8. **Fig. S8:** SEM images of tensile fractography of LVB_0_20 min micro/nanocellulose filled epoxy sheet (**a**, **b**, **c** - 45º) - (**d**, **e**, **f** - 90º) tested at 80 ºC
9. **Fig. S9:** SEM images of tensile fractography of SMC_0.1_60 min micro/nanocellulose filled epoxy sheet (**a**, **b**, **c** - 45º) - (**d**, **e**, **f** - 90º) tested at 80 ºC
10. **Fig. S10:** Contact angle measurements on top and bottom surfaces of micro/nanocellulose filled epoxy sheets.
11. **Fig. S11**: Stereomicroscopy image after 30 days of biodegradability on SMC sheet.
12. **Table S1:** Thermogravimetric analysis of micro/nanocellulose filled epoxy sheets, pure epoxy, and micro/nanocellulose sheets
13. **Table S2:** Water absorptivity on micro/nanocellulose filled epoxy sheets.

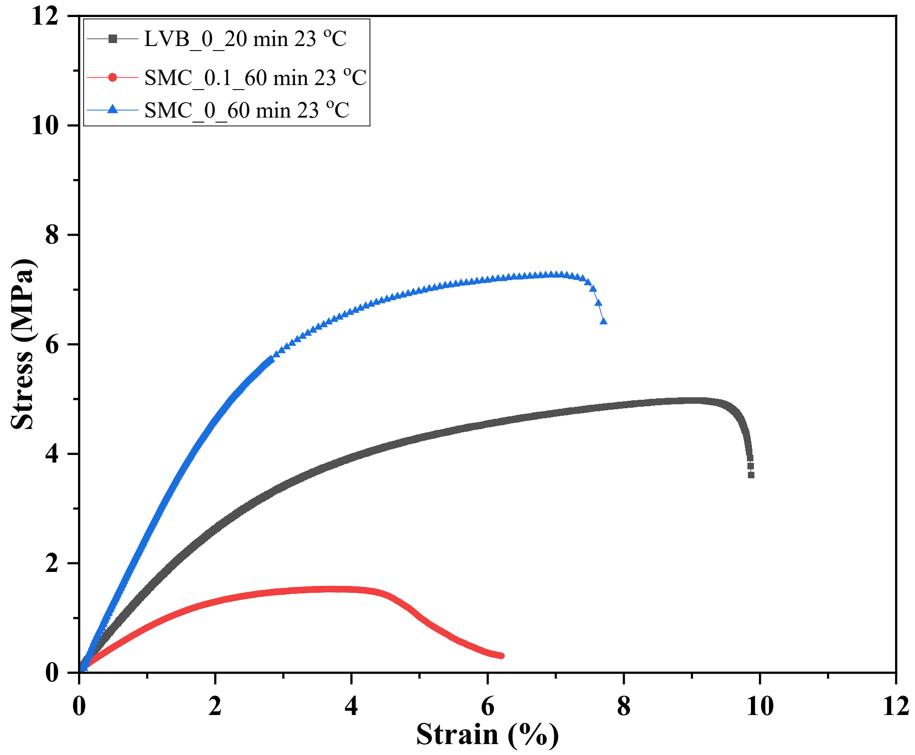

Fig. S1: Tensile stress-strain curves of micro/nanocellulose sheets tested at 23 °C

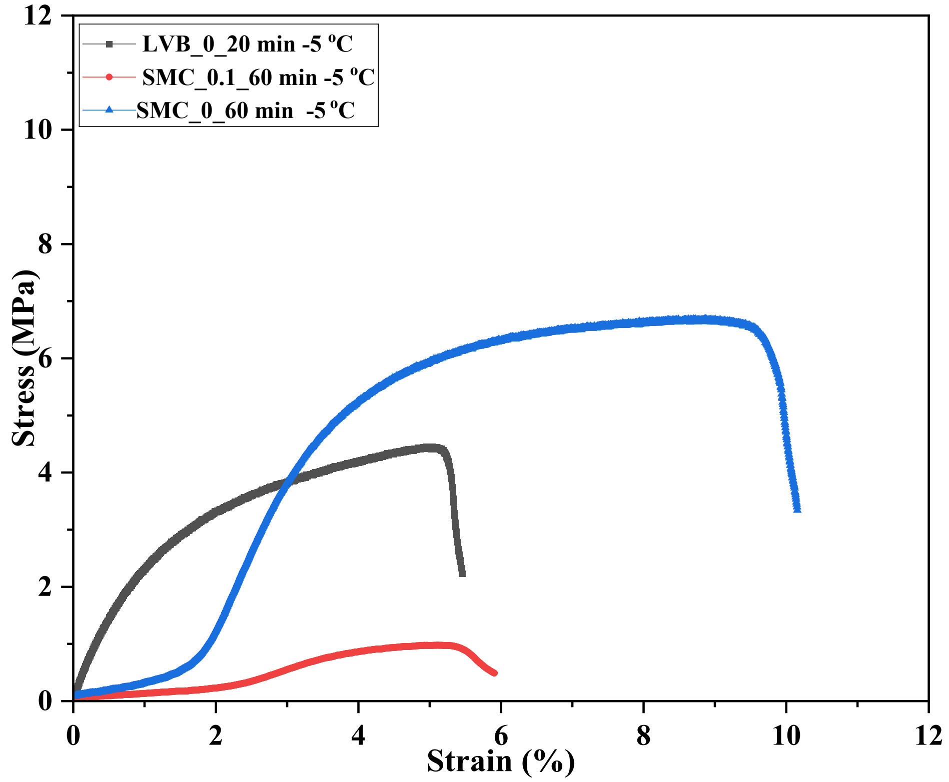

Fig. S2: Tensile stress-strain curves of micro/nanocellulose sheets tested at -5 °C

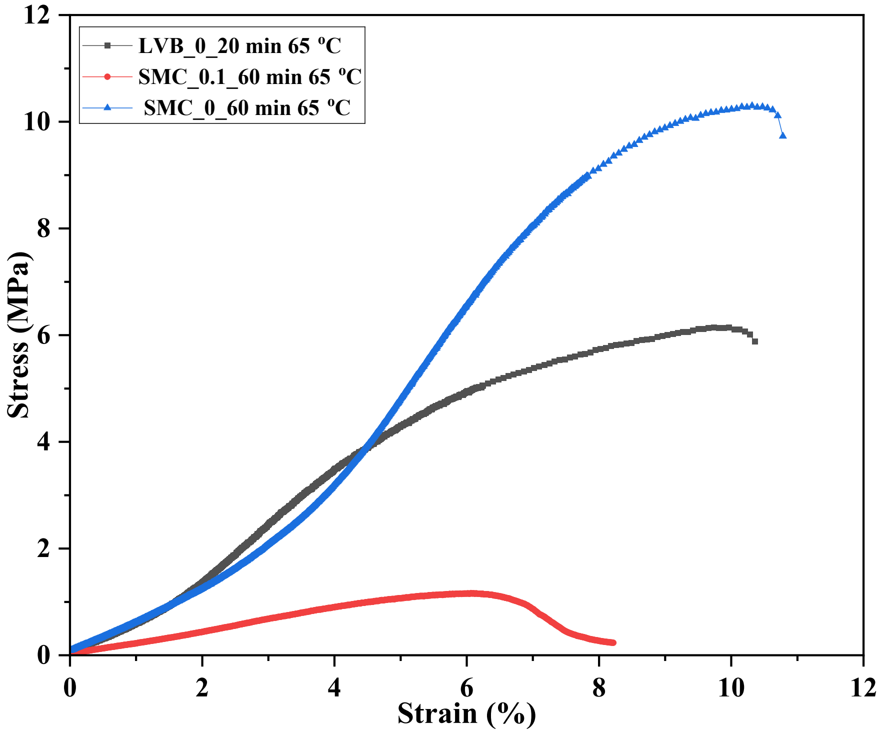

Fig. S3: Tensile stress-strain curves of micro/nanocellulose sheets tested at 65 °C

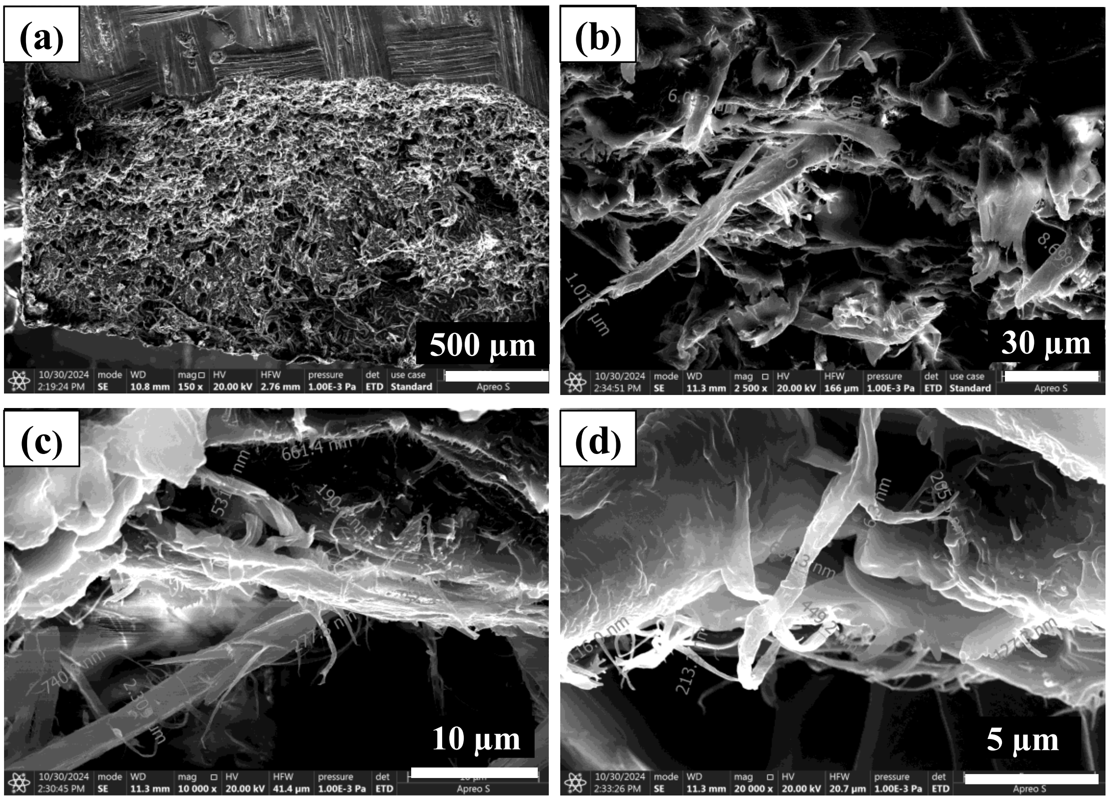

Fig. S4: SEM images of tensile fractography of LVB_0_20 min_ epoxy sheet tested at RT

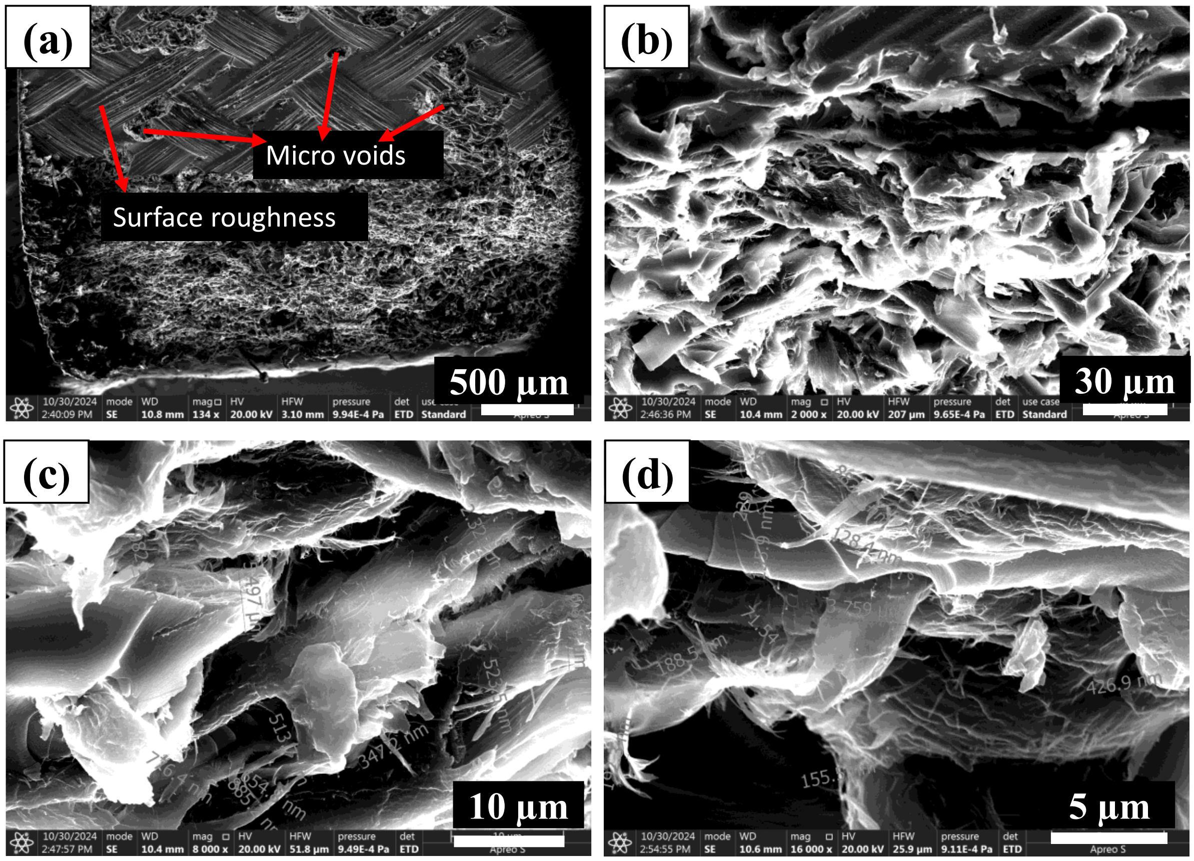

Fig. S5: SEM images of tensile fractography of SMC_0.1_60 min_ epoxy sheet tested at RT (23 ºC)

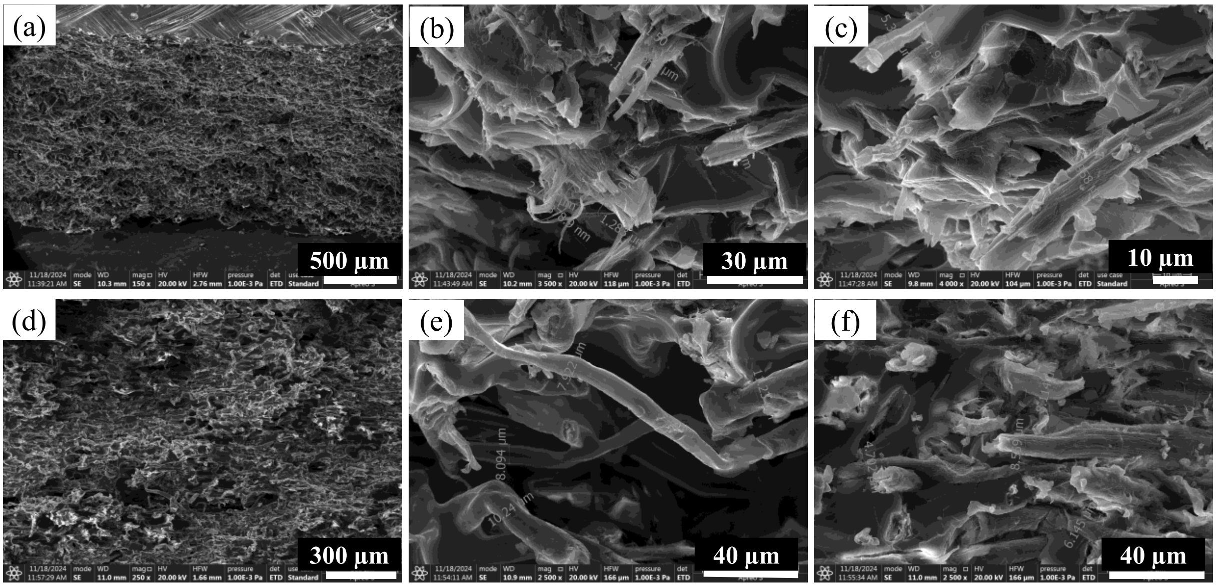

Fig. S6: SEM images of tensile fractography of LVB_0_20 min_epoxy sheet (**a**, **b**, **c** -45º) - (**d**, **e**, **f** – 90º) tested at -20 ºC

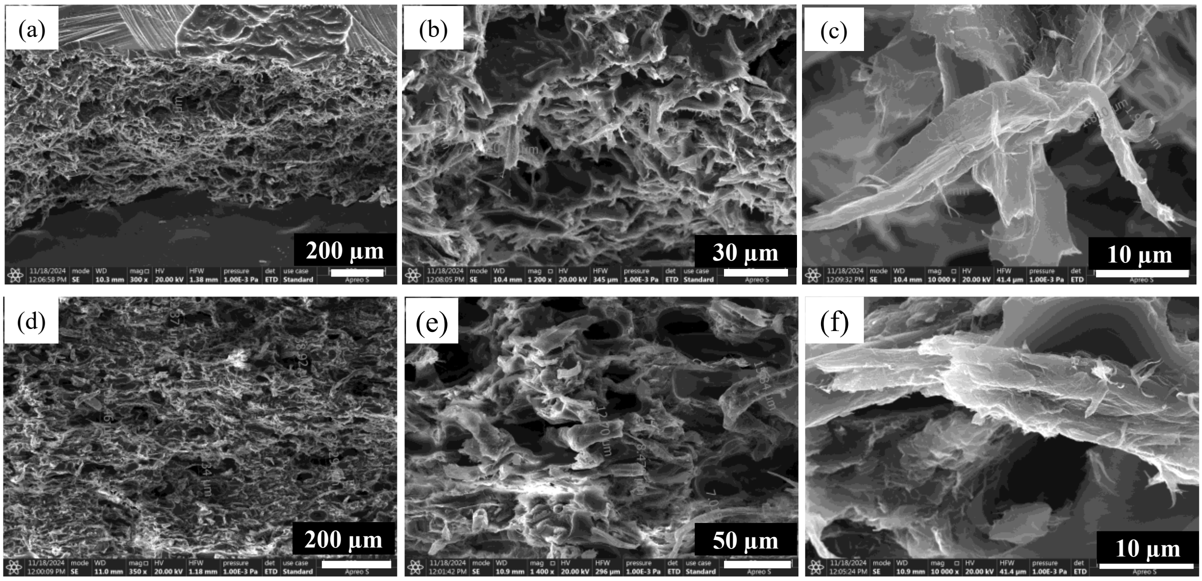

Fig. S7: SEM images of tensile fractography of SMC_0.1_60 min_epoxy sheet (**a**, **b**, **c** – 45º) - (**d**, **e**, **f** – 90º) tested at -20 ºC

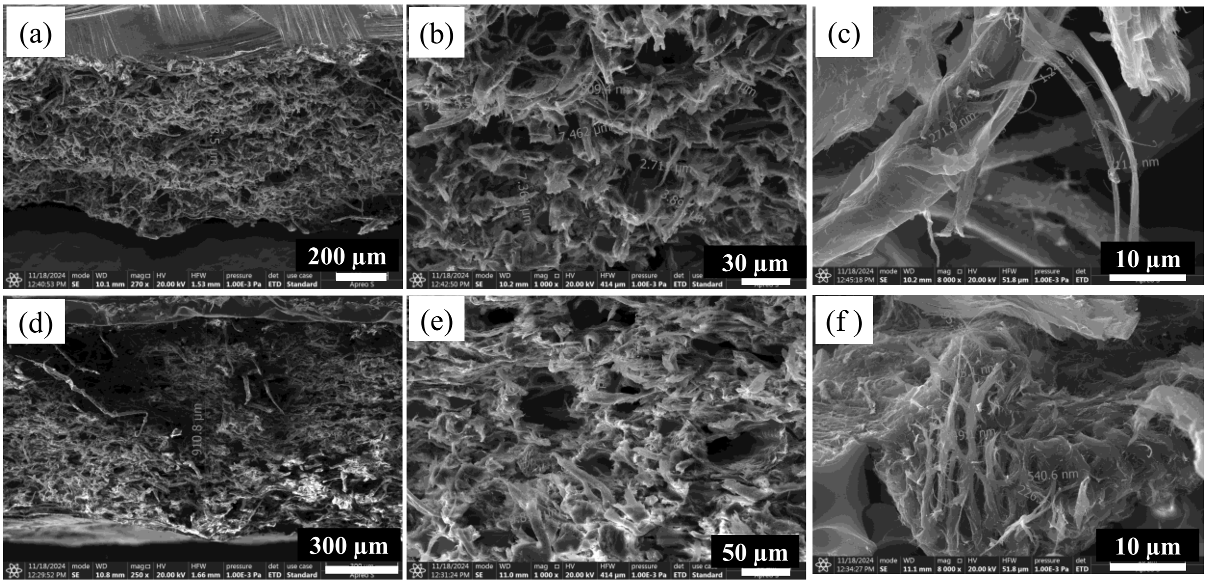

Fig. S8: SEM images of tensile fractography of LVB_0_20 min micro/nanocellulose filled epoxy sheet (**a**, **b**, **c** - 45º) - (**d**, **e**, **f** - 90º) tested at 80 ºC

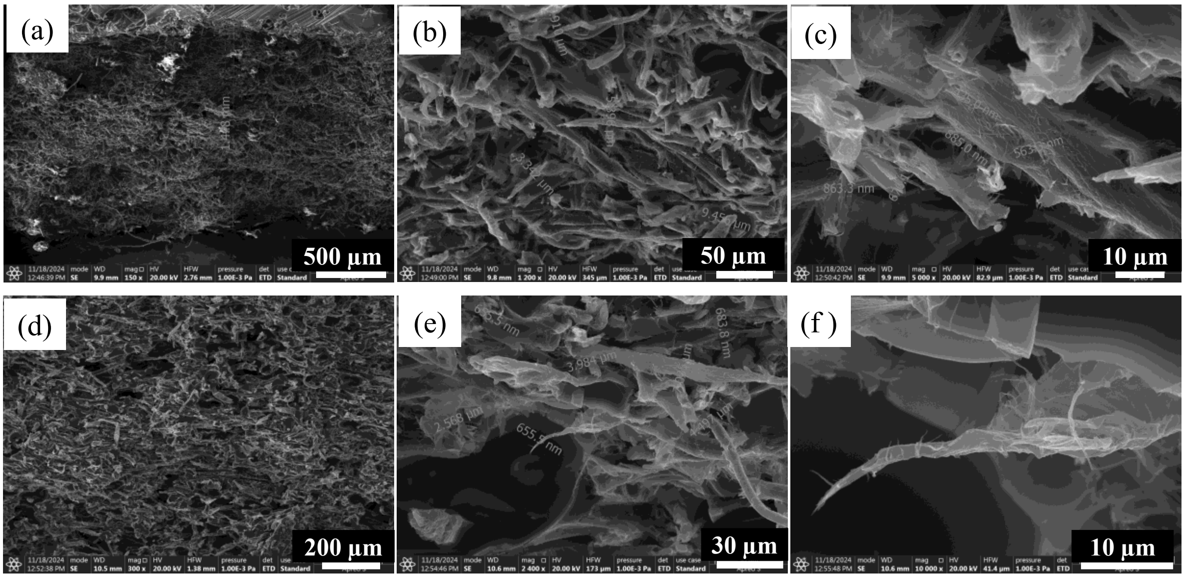

Fig. S9: SEM images of tensile fractography of SMC_0.1_60 min micro/nanocellulose filled epoxy sheet (**a**, **b**, **c** - 45°) - (**d**, **e**, **f** - 90°) tested at 80 °C

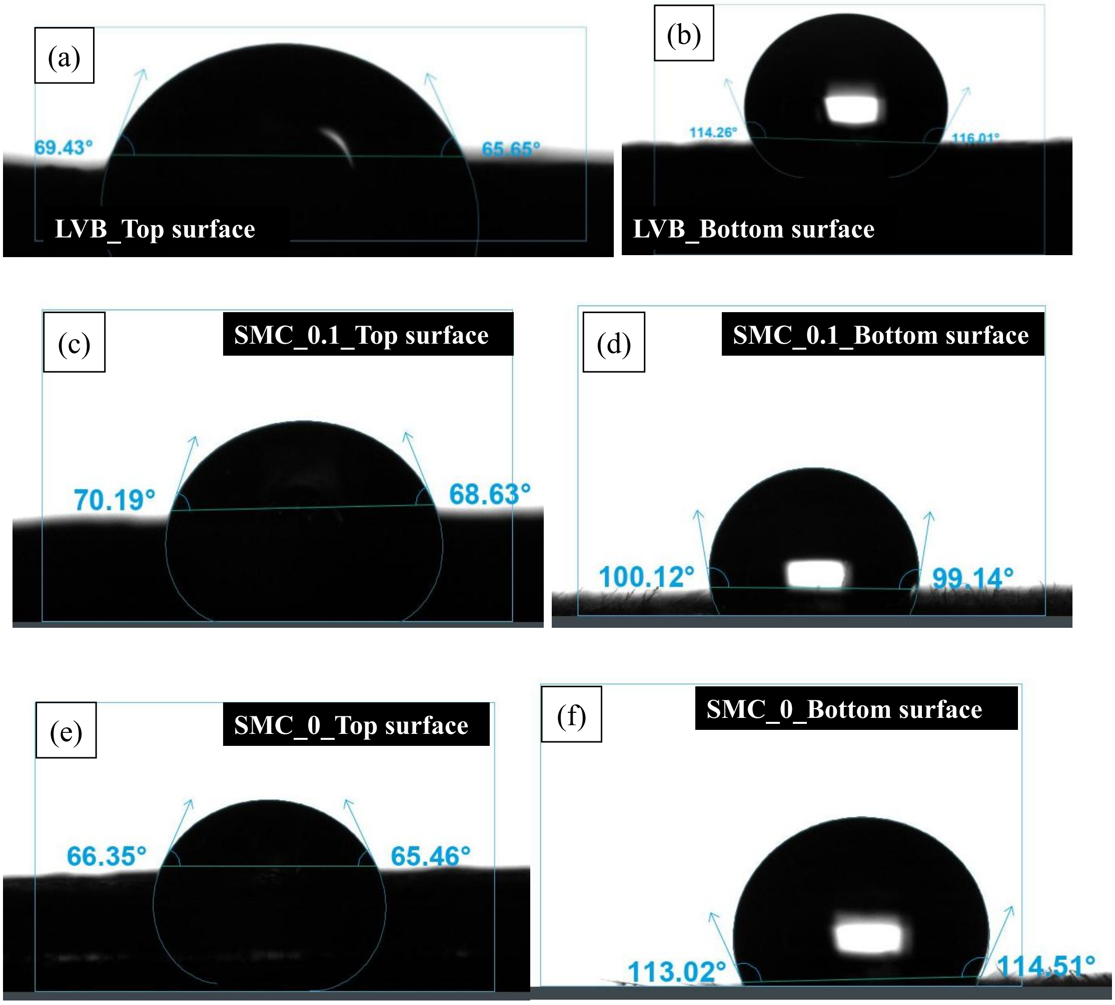

Fig. S10: Contact angle measurements on top and bottom surfaces of micro/nanocellulose filled epoxy sheets.

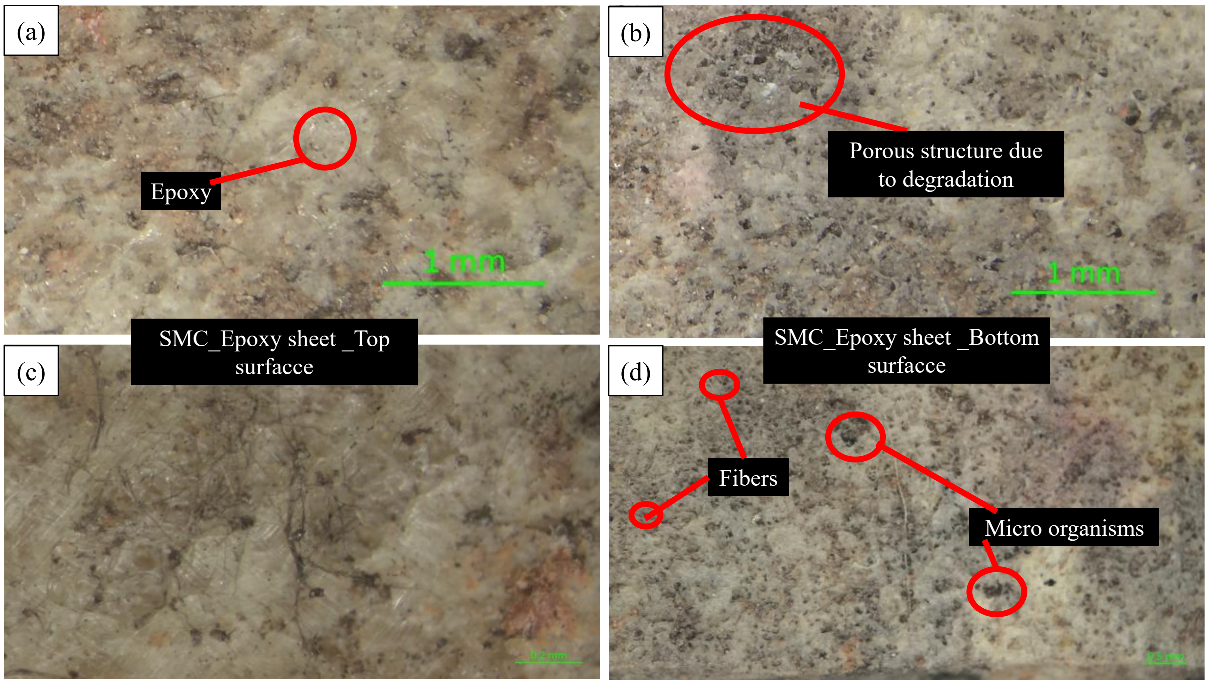

Fig. S11: Stereomicroscopy image after 30 days of biodegradability on SMC sheet.

Table S1: Thermogravimetric analysis of micro/nanocellulose filled epoxy sheets, pure epoxy, and micro/nanocellulose sheets

S. No	Material	Zone 1- till 220 °C			Zone 2-till 430 °C			Zone 3- till 600 °C			Cumulative weight loss (%) up to 600 °C
		T_i (°C)	T_f (°C)	Weight loss (%)	T_i (°C)	T_f (°C)	Weight loss (%)	T_i (°C)	T_f (°C)	Weight loss (%)	
1	LVB_0_20 min_epoxy sheet	31.4	220	1.98	220	430	58.23	430	600	33.01	93.22
2	SMC_0.1_60 min_ epoxy sheet	31.4	220	1.16	220	430	55.99	430	600	37.37	94.52
4	SMC_0_60 min_epoxy sheet	31.4	220	1.91	220	430	60.17	430	600	32.89	94.97
5	Epoxy only	31.4	220	2.04	220	430	68.73	430	600	8.74	79.51
6	Nanocellulose only	31.4	220	4.40	220	430	80.54	430	600	10.23	95.17

Table S2: Water absorptivity on micro/nanocellulose filled epoxy sheets.

LVB/SMC_epoxy sheet	Water absorptivity in Wt% (25 °C, 98% rh)	Water absorptivity in Wt% (40 °C, 98% rh)	Water absorptivity in Wt% (60 °C, 98% rh)
LVB_20 min	3.1	2.3	2.9
SMC_0.1_60 min	1.5	1.5	1.6
SMC_0_60 min	4.0	4.0	4.5

END OF THE DOCUMENT
